# Synthesis and Antisense Properties of 2′β-F-Arabinouridine Modified Oligonucleotides with 4′-*C*-OMe Substituent

**DOI:** 10.3390/molecules23092374

**Published:** 2018-09-17

**Authors:** Xiao-Yang He, Jing Wang, Dan-Dan Lu, Sheng-Qi Wang

**Affiliations:** 1 Beijing Institute of Radiation Medicine, Beijing 100850, China; lu_dandan@163.com; 2 School of Pharmaceutical Sciences, Zhengzhou University, Zhengzhou 450001, China; anna_wj@163.com

**Keywords:** fluorine, pseudohydrogen bond, arabinonucleotide, chemical modification

## Abstract

A novel 2′-F,4′-*C*-OMe–arabinouridine (araU) was successfully synthesized and introduced into oligonucleotides. The oligonucleotide containing 2′-F,4′-*C*-OMe–araU exhibited improved nuclease resistance and RNA hybridizing selective ability relative to 2′-F–araU. In particular, when 2′-F,4′-*C*-OMe–araU inserted into C–H⋯F–C bonding-favorable 5′–uridine–purine–3′ steps, the modified oligonucleotide showed remarkable binding affinity and selectivity to RNA complements. Thus, 2′-F,4′-*C*-OMe–araU has valuable antisense properties and can be used as novel chemical modification for antisense therapeutic strategy.

## 1. Introduction

Chemical modification is essential to the efficient therapeutic application of antisense oligonucleotides. However, research on novel chemically modified nucleotides with optimal binding affinity, nuclease stability, and other desired oligonucleotides properties is still necessary [1,2,3]. Among numerous modifications available to date, fluorinated nucleotide derivatives are intensively investigated and play a privileged role in oligonucleotides [4,5,6]. Fluorine is small, with size similar to that of hydrogen; however, due to its higher electronegativity and low polarizability, fluorine can profoundly impact physicochemical and biological properties, such as hydrogen bonding, stability, and lipophilicity [7,8]. Specifically, fluorinated modification on the sugar moiety of the nucleotides can pre-organize sugar puckering, and this behavior is closely related to the binding affinity of oligonucleotides [8,9,10,11].

Thereof, as a simple and obvious fluorinated nucleotide on the sugar ring, 2′-deoxy-2′-fluoroarabinonucleic acid (2′-F–ANA) is widely applied in antisense, small interfering RNA (siRNA), riboenzyme, micro RNA (miRNA), aptamer-based oligonucleotides [12]. Once incorporated into the DNA strand of DNA–RNA hybrids, 2′-F–ANA can lead to an increase in the binding affinity compared to its 2′-OH congeners. Nevertheless, this thermal stabilizing effect is largely attributed to the formation of the C–H⋯F–C pseudohydrogen bonds between 2′F and purine H8 of the 3′-proximal nucleotide at 5′–pyrimidine–purine–3′ steps, where the base-stacking geometry is pre-organized to optimize the interaction without incurring unfavorable contacts [9,13,14]. In addition, unlike its epimeric 2′α-F–RNA, 2′-F–ANA is a DNA mimic. The presence of 2′β-F is believed to promote an [O4′–C1′–C2′–F2′] gauche interaction to steer the sugar puckers toward the C2′-*endo*/C4′-*endo* (southeast) conformation. Nevertheless, 2′-F–ANA can be sufficiently flexible to adopt a preferred north conformation due to the limited steric interactions of the fluorine atom. Moreover, 2′-F–ANA is found to be generally more stable to snake venom phosphodiesterase (SVPDE) than corresponding DNA and 2′-F–RNA [12]. Inspired by these intriguing features of 2′-F–ANA, we set out to design a series of 2′-F–ANA-based chemically modified nucleotides.

**Figure 1:**
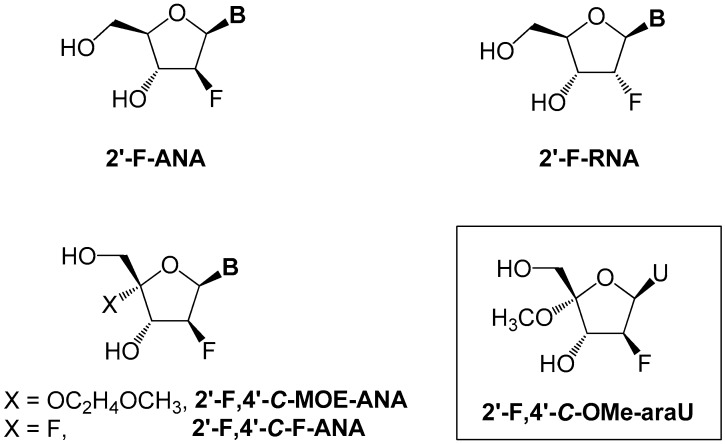
Structure of fluorinated nucleotide 2′-F–arabinonucleic acid (ANA), 2′-F–RNA, selected 4′-substituted 2′-F–ANA derivatives, and 2′-F,4′-*C*-OMe–arabinouridine (araU) designed in the present study. B = nucleobase, U = uracil-1-yl.

We chose to introduce an alkoxy group into the C4′-position of 2′-F–ANA, thus designing a novel bifunctional 4′-*C*-alkoxy-2′-F–arabinonucleotide derivative as presented in Figure 1. It is reported that the strong anomeric effect of the exocyclic oxygen atom attached to the C4′ carbon can drive the sugar part of 2′-deoxynucleotides toward the RNA-like C3′-*endo* (north) conformation. This conformational behavior is highly beneficial to the binding affinity to complementary RNA [15,16]. Moreover, Damha et al. [17] demonstrated that introduction of 4′-F into 2′-F–araU switched the preferred sugar conformation from DNA-like to RNA-like by the similar anomeric effect of 4′-F. Hence, it was predicted that the 4′-*C*-alkoxy-2′-F–arabinonucleotide derivatives would also pre-organize the sugar ring toward a north conformation, and enhance the hybridization properties for antisense oligonucleotides. Moreover, C4′ modifications on the ribose moiety, such as 4′-S [11], 4′-*C*-OMe [15,16], and 4′-*C*-aminomethyl [10,18], are reliable strategies to increase nuclease resistance. In particular, the C4′-modified 2′-F–uridine derivative, 2′-F,4′-*C*α-OMe–uridine, which was recently reported by Damha et al. [19], was able to increase nuclease resistance due to the close proximity between 4′-OMe substituent and the vicinal 5′- and 3′-phosphate group. Thus, we anticipated that the 4′-*C*-alkoxy-2′-F–arabinonucleotide derivative would retain the C–H⋯F–C pseudohydrogen bonding ability, and, despite the sugar puckering changes, would improve the therapeutic properties relative to 2′-F–ANA.

Aiming to develop the 4′-*C*-alkoxy-2′-F–arabinonucleotide derivative for antisense application, we previously constructed and evaluated a bifunctional modification 2′-deoxy-2′-F,4′-*C*-(2-methoxyethoxy)–arabinouridine (2′-F,4′-*C*-MOE–araU) [20]. We found that the oligonucleotides modified with 2′-F,4′-*C*-MOE–araU, especially in the context of C–H⋯F–C bonding-favorable 5′–uridine–purine–3′ steps, showed significant RNA selectivity and duplex stability when hybridized to RNA complements. This finding suggested that combination of 4′-*C*-alkoxy modification and pseudohydrogen bond of 2′β-F is compatible. However, 2′-F,4′-*C*-MOE–araU at the 3′-terminal of the oligonucleotide was found to decrease nuclease resistance ability compared to corresponding 2′-F–araU. These results prompted us to develop novel 4′-*C*-alkoxy substituents to further explore the potential of 2′-F–ANA. Herein, we report the synthesis and antisense properties of 2′-deoxy-2′-F,4′-*C*-methoxy arabinouridine (2′-F,4′-*C*-OMe–araU) as a novel 2′-F–ANA derivative with C4′-methoxy substitution.

## 2. Results and Discussion

### 2.1. Chemistry

**Scheme 1:**
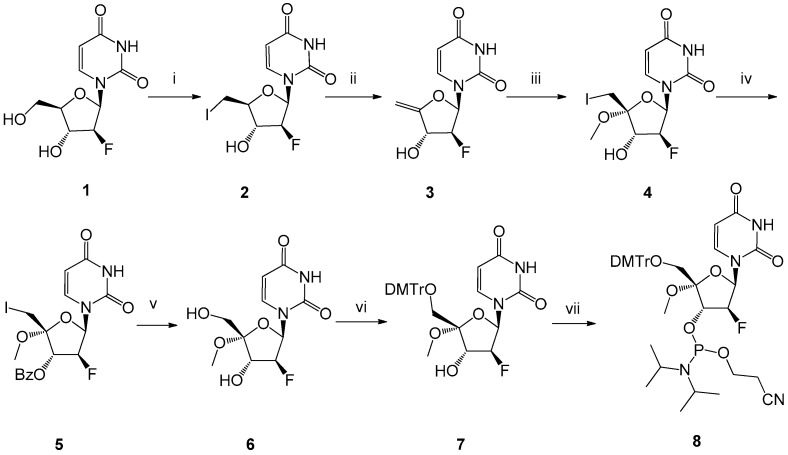
The general synthesis procedure of 2′-F,4′-*C*-OMe–araU phosphoramidite **8**. Reagents and conditions: (**i**) imidazole, Ph_3_P, I_2_, tetrahydrofuran (THF), 0 °C–room temperature (rt), 4 h, 78%; (**ii**) NaOMe–MeOH, reflux, 3 h, 85%; (**iii**) methanol, PbCO_3_, I_2_, THF (dry), 0 °C–rt, 3 h, 90%; (**iv**) benzoyl chloride (BzCl), pyridine (dry), dichloromethane (DCM), 0 °C–rt, 3 h, 95%; (**v**) (a) *m*-chloroperbenzoic acid (*m*-CPBA), DCM/H_2_O, 0 °C–rt, 3 h, (b) NH_3_–MeOH, rt, 12 h, 64% two steps; (**vi**) dimethoxytrityl chloride (DMTrCl), pyridine (dry), rt, 12 h, 81%; (**vii**) 2-cyanoethyl *N*,*N*,*N*′,*N*′-tetraisopropylphosphoramidite, 1*H*-tetrazole, DCM (dry), room temperature, 3 h, 74%.

The synthesis of the 2′-F,4′-*C*-OMe–araU derivative was performed as depicted in Scheme 1. According to the reported method [21], treatment of 2′-F–araU **1** with I_2_/Ph_3_P in tetrahydrofuran (THF), followed by elimination with sodium methoxide (NaOMe) in methanol, readily generated the 4′-methylene **3**. Treatment of **3** with iodine/methanol in the presence of PbCO_3_ yielded the 4′-methoxy analog **4**; in this preparation, the 4′-OMe substituent was introduced by opening the transient iodonium ion of 4′-methylene nucleoside **3** with methanol (MeOH) in a regio- and stereo-specific manner. Benzoylation of **4**, followed by treatment with *m*-chloroperbenzoic acid (*m*-CPBA) in water-saturated dichloromethane and subsequent hydrolysis in NH_3_–MeOH afforded the target nucleoside monomer, 2′-F,4′-*C*-OMe–araU **6**. Next, the treatment of **6** with dimethoxytrityl chloride (DMTrCl) in pyridine generated tritylated **7**. Then, phosphitylation of **7** with 2-cyanoethyl *N*,*N*,*N*′,*N*′-tetraisopropylphosphoramidite in the presence of 1*H*-tetrazole gave phosphoramidite **8** as a standard building block for automated oligonucleotide synthesis. Finally, **8** was incorporated into oligonucleotides on an automated DNA synthesizer using 5-ethylthio-1*H*-tetrazole as an activator with a prolonged coupling time of 6 min. After cleavage from the solid support and deprotection, all corresponding sequences were purified by reversed-phase (RP) HPLC chromatography and validated by mass spectrometry (Supplementary Materials, Table S1).

### 2.2. Hybridization Properties

**Figure 2:**
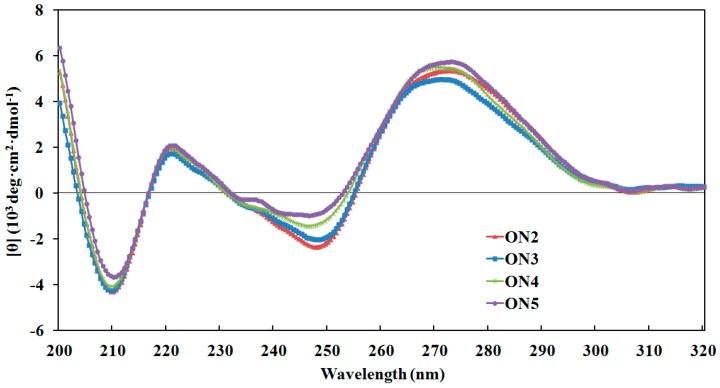
The circular dichroism (CD) spectra of duplexes formed by the modified oligonucleotides **ON2**–**ON5** with RNA complements. Spectra were recorded three times independently at 20 °C after annealing the duplexes under the same condition described in ultraviolet (UV) melting experiments.

The duplex-forming abilities of 2′-F,4′-*C*-OMe–araU and 2′-F–araU modified oligonucleotides with their complementary single-stranded RNA and DNA (ssRNA and ssDNA) were evaluated by ultraviolet (UV)-thermal denaturation experiments and their properties were compared with those of the corresponding natural DNA. As can be seen in Table 1, the melting temperature (*T*_m_) value of the 2′-F,4′-*C*-OMe–araU modified oligonucleotide **2** (**ON2**)–RNA hybrid was found to be slightly higher than that of the 2′-F–araU modified **ON3**–RNA hybrid when inserted at the same position of the oligonucleotides. In contrast, the thermal stability of the duplex formed by **ON2** with the DNA complement was lower than that of **ON3**–DNA hybrid. As a result, in **ON2** and **ON3**, the differences in Δ*T*_m_ values with RNA (Δ*T*_m_ (RNA)) and DNA (Δ*T*_m_ (DNA)) were +2.8 °C and −0.7 °C, respectively. Thus, 2′-F,4′-*C*-OMe–araU modified **ON2** had enhanced RNA selectivity relative to 2′-F–araU. In addition, the 2′-F,4′-*C*-OMe–araU modification contributed to RNA selectivity in **ON4**–**5**, **ON7**–**8**, and **ON10**–**11**. Since the RNA-selective hybridization ability was highly related to the conformational preorganization of the sugar, we investigated the ^1^H–^1^H *J*-coupling constants of H2″/H3′ and H1′/F2′ to determine the sugar conformation. According to the Karplus equation’s prediction [17], a northern conformer will have a large ^3^*J*_H2″H3′_ coupling constant, whereas a southern conformer will have a large ^3^*J*_H1′F2′_ value. The experimental ^3^*J*_H2″H3′_ and ^3^*J*_H1′F2′_ values of 2′-F,4′-*C*-OMe–araU in D_2_O were 5.6 and 9.0 Hz, respectively, whereas those for 2′-F–araU were 3.0 and 17.5 Hz, respectively (Supplementary Materials, Table S2). These data indicated the high preference for north pucker in 2′-F,4′-*C*-OMe–araU, as previously predicted. Therefore, we concluded that the 4′-*C*-OMe in 2′-F,4′-*C*-OMe–araU was able to overcome the gauche effect of the 2′βF, which generally steers the sugar pucker toward a southeast conformation, and able to impart the conformation preference toward the north pucker through the strong anomeric effect. This interaction improves the hybridization property of the modified oligonucleotides. Moreover, the circular dichroism (CD) spectra showed that oligonucleotides with either single or multiple 2′-F,4′-*C*-OMe–araU modifications (**ON2**, **ON4**–**5**) were able to form canonical A-form duplexes with the RNA complement (Figure 2), suggesting that the conformation pre-organization on the arabino-sugar, induced by C4′-OMe substitution, would facilitate the duplex-forming ability to the RNA complement, which would exert a positive effect on the binding affinity of antisense oligonucleotides.

However, the formation of modified oligonucleotides with double or triple 2′-F,4′-*C*-OMe–araU led to significant duplex destabilization. The *T*_m_ values of oligonucleotides **ON4** and **ON5** were lower than those of natural DNA **ON1** after hybridization to both RNA and DNA complements. Since the 2′-F,4′-*C*-OMe–araU sugar puckering prefers the north conformation, it is likely that this would lead to an unfavorable inter-nucleoside contact between 2′F and the C5 methyl group of the 3′-adjacent thymidine, and result in a destabilization effect on the duplex formation. Therefore, the hybridization ability of **ON4** and **ON5** with those RNA and DNA complements is decreased due to the repulsive interaction between 2′F and the C5 methyl group of the 3′-adjacent thymidine, although the conformation pre-organization of the sugar is beneficial to duplex formation. On the other hand, considering that the thermal stabilizing effect of 2′-F–ANA is largely attributed to the C–H⋯F–C pseudohydrogen bonding interaction with the 3′-adjacent purine, to further assess the impact of 2′-F,4′-*C*-OMe–araU modification, we designed oligonucleotides **ON7**–**8** and **ON10**–**11** with a purine nucleotide (G or A) in the center of the sequence and flanked with the 2′-F,4′-*C*-OMe–araU monomer on either the 5′- or 3′-position of the central purine, respectively. As shown in Table 1, in the case of 2′-F,4′-*C*-OMe–araU modified **ON7** and **ON10** with preferable 5′–uridine–purine–3′ steps (**XG** and **XA**, respectively), the *T*_m_ values of duplexes formed with RNA complements were substantially higher than those of the duplexes formed by the natural DNAs **ON6** and **ON9** with RNA complements; the corresponding Δ*T*_m_ values were 1.6 °C and 2.6 °C. In contrast, **ON8** and **ON11**, whose 2′-F,4′-*C*-OMe–araU modifications were located at the 3′-proximity of the central purine, showed a destabilizing effect when hybridized to RNA complements relative to their counterpart natural DNA–RNA duplexes. These results clearly illustrated that the C–H⋯F–C pseudohydrogen bonding interaction at the favorable 5′–pyridimine–purine–3′ step exerted a remarkably stabilizing effect on the RNA complement. Additionally, compared with the hybridization ability of **ON7** and **ON10** with their DNA complements, 2′-F,4′-*C*-OMe–araU modification at the 5′–uridine–purine–3′ steps showed higher RNA selectivity than those of **ON2**, **ON8**, and **ON11**. The respective differences of Δ*T*_m_ values with RNA (Δ*T*_m_ (RNA)) and with DNA (Δ*T*_m_ (DNA)) were within the range 5–6 °C. Generally, 2′βF in 2′-F,4′-*C*-OMe–araU was still capable of forming a pseudohydrogen bonding interaction with purine at the 5′–uridine–purine–3′ steps, which remarkably increased the binding affinity, despite the change in sugar conformation.

### 2.3. Nuclease Stability

**Figure 3:**
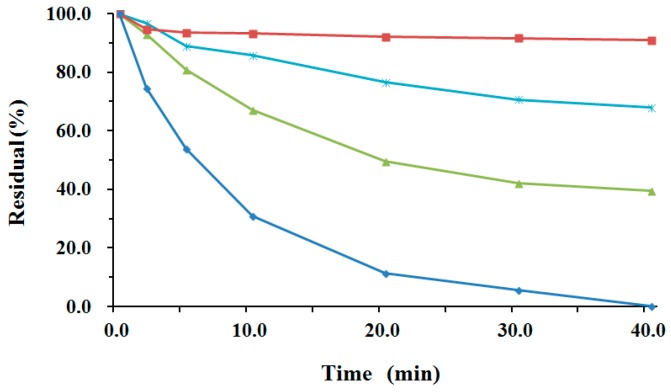
Enzymatic stability of 5′–d(TTTTTTTT***U***T)–3′ against snake venom phosphodiesterase (SVPDE). ***U*** = 2′-F,4′-*C*-OMe–araU (**X**, **ON12**, cross), 2′-F–araU (**Z**, **ON13**, triangle), 3′-phosphorothioate-T (**Ts**, **ON14**, rectangle), and natural DNA–dT (**ON15**, diamond). Degradation of oligonucleotides (17.5 pM) by SVPDE (0.02 μg/mL) was performed in a buffer (pH 8.0) containing 10 mM MgCl_2_, 50 mM Tris-HCl at 37 °C.

The nuclease stability of the T-decamer oligonucleotide **ON12** containing 2′-F,4′-*C*-OMe–araU at the second position of the 3′-terminus was investigated using snake venom phosphodiesterase (SVPDE) as a 3′-exonuclease, and was then compared with that of 2′-F–araU modified, 3′-phophorothioate-T and natural oligonucleotides, **ON13**–**ON15** (Figure 3). Under the conditions used in this experiment, approximately 75% oligonucleotide **ON12** was still retained after 40 min of incubation. As a result, the 2′-F,4′-*C*-OMe–araU modification provided greater stability toward the 3′-exonuclease compared to corresponding 2′-F–araU modified **ON13** and the unmodified **ON15**. Since the increased nuclease resistance was associated mainly with the close vicinity of the hydrophobic moiety and neighboring phosphates [19], it appeared that the 4′-*C*-OMe substituent was the ideal group for promoting the nuclease resistance ability of 2′-F–ANA.

## 3. Conclusions

In conclusion, a novel 2′-F,4′-*C*-OMe–araU was successfully synthesized and incorporated into oligonucleotides. The modified oligonucleotides showed enhanced RNA-selective hybridizing ability relative to 2′-F–araU due to the switch of sugar pucker. It is noteworthy that whether single- or multi-modified, these oligonucleotides could form a typical A conformation with complementary RNA. Meanwhile, the impact of 2′-F,4′-*C*-OMe–araU on duplex thermal stability was sequence-dependent, and oligonucleotides with C–H⋯F–C bonding-favorable 5′–uridine–purine–3′ steps had the strongest binding affinity to ssRNA. In addition, the stability against nuclease degradation of the oligonucleotides containing 2′-F,4′-*C*-OMe–araU was greater than that of those containing 2′-F–araU. These investigations revealed that the introduction of 4′-*C*-OMe into 2′-F–araU had synergistic effects on hybridization ability with ssRNA and the nuclease resistance, suggesting that 2′-F,4′-*C*-OMe–araU has valuable antisense properties and can be reasonably applied in the development of antisense oligonucleotides.

## 4. General Experimental Procedure

All chemical reagents were commercially available and used without any further purification unless otherwise mentioned. Anhydrous dichloromethane (DCM), tetrahydrofuran (THF), pyridine (Pyr), and acetonitrile (ACN) were distilled from CaH_2_. Reactions involving air and/or those that were moisture-sensitive were performed under nitrogen atmosphere, and the reaction process was monitored by analytical thin-layer chromatography (TLC; silica gel GF254). Flash-column chromatography was carried out with Teledyne Isco Combiflash Rf200 purification (Teledyne Isco Inc., Lincoln, NE, USA). ^1^H-NMR (400 MHz), ^19^F-NMR (376 MHz), and ^31^P-NMR (162 MHz) were recorded on a JEOL JNM-ECA-400 spectrometer (JOEL Ltd., Tokyo, Japan) with tetramethylsilane as the internal standard for ^1^H-NMR spectra, and CFCl_3_ and 85% H_3_PO_4_ as the external standard for ^19^F-NMR and ^31^P-NMR spectra, respectively. Mass spectra of small molecules were measured on an Agilent 1260-G6230A mass spectrometer (Agilent Technologies Inc., Santa Clara, CA, USA), mass spectra of oligonucleotides were recorded on a Bruker Daltonics Ultraflex^TM^ MALDI-TOF/TOF spectrometer (Bruker Daltonics Inc., Billerica, MA, USA) or a Thermo-Finnigan liquid chromatography quadrupole (LCQ) Ion Trap spectrometer (Thermo Fisher Scientific Inc., MA, USA). Circular dichroism (CD) spectra were performed on a Bio-logic MOS-450 CD spectrometer (Bio-Logic Inc., Claix, France). UV melting experiments were carried out on a SHIMADZU UV-1800 spectrometer (Shimadzu Corp., Kyoto, Japan) equipped with a *T*_m_ analysis accessory.

### 4.1. Chemistry

#### 4.1.1. 2′,5′-Dideoxy-5′-iodo-2′-fluoro-β-d-arabinouridine (**2**)

To a mixture of 2′-F–araU **1** (10.0 g, 40.62 mmol), Ph_3_P (15.98 g, 60.93 mmol) and imidazole (5.53 g, 81.24 mmol) in 200 mL on THF were added dropwise to a solution of I_2_ (15.42 g, 60.93 mmol) in 100 mL of THF. After addition, the reaction mixture was stirred at room temperature for 4 h. Saturated Na_2_SO_3_ solution was added, and the mixture was extracted with ethyl acetate (EtOAc), washed with water and brine, and dried with Na_2_SO_4_. After concentration in vacuo, the residue was purified by flash chromatography (gradient eluent: MeOH/DCM, 0–10% *v*/*v*) to afford the 5′-iodination compound **2** as a pale-yellow solid, yield 78%. ^1^H-NMR (400 MHz, CD_3_OD) δ 7.71 (dd, *J* = 8.1, 1.9 Hz, 1H, H-6), 6.21 (dd, *J* = 20.0, 3.4 Hz, 1H, H-1′), 5.71 (d, *J* = 8.1 Hz, 1H, H-5), 5.02 (ddd, *J* = 51.8, 3.4, 1.8 Hz, 1H, H-2′), 4.29 (ddd, *J* = 17.3, 3.2, 1.8 Hz, 1H, H-3′), 3.99 (td, *J* = 6.2, 3.4 Hz, 1H, H-4′), 3.55–3.43 (m, 2H, H-5′); electrospray ionization (ESI)-MS (*m*/*z*) 357.1 [M + H]^+^.

#### 4.1.2. 2′,5′-Dideoxy-2′-fluoro-4′-methylene-β-d-arabinouridine (**3**)

To a solution of compound **2** (11.5 g, 32.31 mmol) in 100 mL of MeOH, 50% NaOMe (13.96 g, 129.24 mmol) was added under nitrogen atmosphere. The reaction mixture was then heated to reflux for 4 h. After cooling to room temperature, the reaction mixture was neutralized with acetic acid (HOAc) to pH 7–8, After the removal of solvent in vacuo, the residue was purified by flash chromatography (gradient eluent: MeOH/DCM, 0–10% *v*/*v*) to afford the 4′-methylene **3** as a white solid, yield 85%. ^1^H-NMR (400 MHz, CD_3_OD) δ 7.47 (dd, *J* = 8.2, 2.1 Hz, 1H, H-6), 6.51 (dd, *J* = 19.5, 3.2 Hz, 1H, H-1′), 5.72 (d, *J* = 8.2 Hz, 1H, H-5), 5.11–4.96 (m, 1H, H-2′), 4.73–4.63 (m, 2H, H-3′ and =CH_2_), 4.47 (d, *J* = 2.3 Hz, 1H, =CH_2_); ESI-MS (*m*/*z*) 229.2 [M + H]^+^.

#### 4.1.3. 2′,5′-Dideoxy-2′-fluoro-5′-iodo-4′-*C*-methoxy-β-d-arabinouridine (**4**)

To a mixture of compound **3** (2.8 g, 12.27 mmol) in 100 mL of THF, PbCO_3_ (6.78 g, 24.54 mmol) and anhydrous MeOH (10 mL) were successively added; a solution of I_2_ (6.23 g, 24.54 mmol) in 50 mL of THF was added dropwise at 0 °C. After addition, the reaction mixture was slowly warmed to room temperature and stirred for 3 h, quenched with saturated Na_2_SO_3_ solution, and filtrated with kieselguhr. The filtrate was extracted with EtOAc, washed with water and brine, dried over Na_2_SO_4_, and concentrated in vacuo. The residue was purified by flash chromatography (gradient eluent: MeOH/DCM, 0–10% *v*/*v*) to afford compound **4** as a white solid, yield 90%. ^1^H-NMR (400 MHz, CD_3_OD) δ 7.72 (dd, *J* = 8.2, 1.7 Hz, 1H, H-6), 6.26 (dd, *J* = 12.0, 5.3 Hz, 1H, H-1′), 5.74 (d, *J* = 8.2 Hz, 1H, H-5), 5.21–5.03 (m, 1H, H-2′), 4.55 (dd, *J* = 24.7, 4.5 Hz, 1H, H-3′), 3.80 (d, *J* = 11.5 Hz, 1H, Ha-5′), 3.54 (d, *J* = 11.6 Hz, 1H, Hb-5′), 3.41 (s, 3H, –OCH_3_); ESI-MS (*m*/*z*) 385.0 [M − H]^−^.

#### 4.1.4. 3′-*O*-Benzoyl-2′,5′-dideoxy-2′-fluoro-5′-iodo-4′-*C*-methoxy-β-d-arabinouridine (**5**)

To a solution of compound **4** (2.4 g, 6.22 mmol) and pyridine (1 mL, 12.6 mmol) in 30 mL of anhydrous CH_2_Cl_2_, benzoyl chloride (856 μL, 7.46 mmol) was added dropwise at 0 °C; then, the reaction mixture was warmed slowly to room temperature and stirred for 3 h. The reaction was then quenched with water and extracted with CH_2_Cl_2_, washed with water and brine, dried over MgSO_4_, and concentrated in vacuo. The residue was then purified by flash chromatography (gradient eluent: MeOH/DCM, 0–10% *v*/*v*) to afford the 3′-*O*-benzoylation **5** as a white powder, yield 95%. ^1^H-NMR (400 MHz, CDCl_3_) δ 8.71 (s, 1H, NH), 8.12–8.05 (m, 2H, o-Ar-H), 7.70 (dd, *J* = 8.2, 2.2 Hz, 1H, H-6), 7.63 (t, *J* = 7.4 Hz, 1H, p-Ar-H), 7.49 (t, *J* = 7.7 Hz, 2H, m-Ar-H), 6.41 (dd, *J* = 15.3, 4.7 Hz, 1H, H-1′), 5.87 (m, 2H, H-5 and H-3′), 5.44 (ddd, *J* = 52.9, 4.6, 3.0 Hz, 1H, H-2′), 3.81 (d, *J* = 11.2 Hz, 1H, Ha-5′), 3.59 (d, *J* = 11.2 Hz, 1H, Hb-5′), 3.38 (s, 3H, –OCH_3_); ESI-MS (*m*/*z*) 512.99 [M + Na]^+^.

#### 4.1.5. 2′-Deoxy-2′-fluoro-4′-*C*-methoxy-β-d-arabinouridine (**6**)

To a solution of compound **5** (2.19 g, 4.46 mmol) in 60 mL/15 mL of DCM/H_2_O (4/1, *v*/*v*), 85% *m*-CPBA (3.08 g, 17.83 mmol) was added in portions at 0 °C. The reaction mixture was slowly warmed to room temperature and stirred for 12 h. After the completion monitored by TLC, the reaction was quenched with saturated Na_2_SO_3_ solution and extracted with CH_2_Cl_2_, washed with saturated NaHCO_3_ solution and brine, dried over MgSO_4_, and concentrated in vacuo. The residue was directly added to excess NH_3_–MeOH solution (20 mL) without purification, and the reaction mixture was stirred at room temperature for 12 h. The resulting mixture was concentrated in vacuo and purified by flash chromatography (gradient eluent: MeOH/DCM, 0–15% *v*/*v*) to afford compound **6** as a white solid, yield 64%. ^1^H-NMR (400 MHz, D_2_O) δ 7.68 (dd, *J* = 8.1, 1.3 Hz, 1H, H-6), 6.31 (dd, *J* = 9.0, 5.8 Hz, 1H, H-1′), 5.80 (d, *J* = 8.1 Hz, 1H, H-5), 5.24 (dt, *J* = 54.0, 5.7 Hz, 1H, H-2′), 4.52 (dd, *J* = 24.0, 5.6 Hz, 1H, H-3′), 3.84 (dd, *J* = 12.6, 1.2 Hz, 1H, Ha-5′), 3.72 (d, *J* = 12.6 Hz, 1H, Hb-5′), 3.32 (s, 3H, –OCH_3_); ESI-MS (*m*/*z*) 299.07 [M + Na]^+^.

#### 4.1.6. 2′-Deoxy-2′-fluoro-5′-*O*-(4,4′-dimethoxy)trityl-4′-*C*-methoxy-β-d-arabinouridine (**7**)

To a mixture of compound **6** (400 mg, 1.45 mmol) in 10 mL of anhydrous pyridine, DMTrCl (740 mg, 2.18 mmol) was added under nitrogen atmosphere. The reaction mixture was stirred at room temperature for 12 h and quenched with MeOH. The resulting reaction mixture was concentrated in vacuo, diluted with EtOAc, washed with water and brine, dried over Na_2_SO_4_, and concentrated in vacuo. The residue was purified by flash chromatography (gradient eluent: MeOH/DCM, 0–5% *v*/*v*) to afford target compound **7** as a white foam, yield 81%. ^1^H-NMR (400 MHz, CDCl_3_) δ 7.68 (d, *J* = 8.1 Hz, 1H, H-6), 7.39–7.26 (m, 9H, Ar–H), 6.85 (m, 4H, Ar–H), 6.11 (d, *J* = 17.8 Hz, 1H, H-1′), 5.32 (d, *J* = 8.1 Hz, 1H, H-5), 4.92 (dd, *J* = 53.0, 5.6 Hz, 1H, H-2′), 4.71 (dd, *J* = 21.8, 5.5 Hz, 1H, H-3′), 3.80 (d, *J* = 1.4 Hz, 6H, 2 × –OCH_3_), 3.56 (d, *J* = 10.1 Hz, 1H, Ha-5′), 3.38 (d, *J* = 10.1 Hz, 1H, Hb-5′), 3.24 (s, 3H, –OCH_3_); ESI-MS (*m*/*z*) 601.2 [M + Na]^+^.

#### 4.1.7. 2′-Deoxy-5′-*O*-(4,4′-dimethoxy)trityl-4′-*C*-methoxy-β-d-arabinouridine-3′-*O*-(2-cyanoethyl-*N*,*N*-diisopropyl)phosphoramidite (**8**)

Under nitrogen atmosphere, 5′-*O*-DMTr compound **7** (400 mg, 0.69 mmol) was dissolved in anhydrous CH_2_Cl_2_; then, 1*H*-tetrazole (70 mg, 0.83 mmol) was added followed by 2-cyanoethyl-*N*,*N*,*N*′,*N*′-tetraisopropyl)phosphoramidite (375 mg, 1.04 mmol). The reaction mixture was stirred at room temperature for 3 h. The resulting mixture was extracted with CH_2_Cl_2_, washed with saturated NaHCO_3_ solution, dried over MgSO_4_, and concentrated in vacuo. The residue was purified by flash chromatography (gradient eluent: MeOH/DCM, 0–5% *v*/*v*) to afford phosphoramidite **8** as a white foam, yield 74%. ^31^P-NMR (162 MHz, DMSO-*d*_6_) δ 151.76, 151.43. ^19^F-NMR (376 MHz, DMSO-*d*_6_) δ −197.10, −197.75; ESI-MS (*m*/*z*) 801.3 [M + Na]^+^.

#### 4.1.8. Oligonucleotide Synthesis

RNA was purchased from Guangzhou RiboBio Inc. The syntheses of other ONs used in this study were performed on an automated synthesizer (K&A H-8) using the standard solid-phase phosphoramidate protocol. Each oligonucleotide was synthesized at the 1-μmol scale using T-CPG support. All DNA phosphoramidites were prepared as 0.11 M solutions in acetonitrile (ACN) except the modified monomers as 0.15 M in ACN. Furthermore, 0.3 M 5-ethylthio-1*H*-tetrazole (ETT) in ACN was used as the activator, 3% dichloroacetic (DCA) acid in DCM was used to detritylate, acetic anhydride + lutidine (Cap A) and 1-methylimidazole (Cap B) in THF were used to cap, and 0.1 M I_2_ in THF/Py/H_2_O (80:40:2, *v*/*v*) was used for oxidation. The coupling time was extended to 6 min for the incorporation of the modified analogs. Cleavage from the solid support and removal of the protecting groups were accomplished using concentrated ammonium hydroxide solution (28% NH_3_ aq.) at 55 °C for 8–12 h. The crude oligonucleotides were purified by reverse-phase HPLC with a Kromasil 100-5C18 (250 mm × 4.6 mm) column (buffer A: 100 Mm hexafluoroisopropanol (HFIP), 31.6 mM triethylamine (TEA), 10% *v*/*v* MeOH; buffer B: MeOH). The obtained pure fractions were analyzed for purity by HPLC chromatography, quantified by UV absorbance at 260 nm, and confirmed by MALDI-TOF or ESI mass spectrometry.

### 4.2. Ultraviolet (UV) Melting Experiments

UV melting experiments were carried out by mixing equimolecular amounts of ONs with DNA or RNA complement in 10 mM sodium phosphate buffer (pH 7.2) containing 100 mM NaCl to give a final concentration of 4 μM. The samples were annealed by heating at 95 °C for 5 min followed by slow cooling to room temperature. Absorbance was recorded at 260 nm from 15–90 °C at a rate of 1 °C/min. The melting temperatures (*T*_m_) were calculated as the first-derivative maximum of the melting curve, and determined by averaging at least three independent measurements, which were accurate within ±1 °C.

### 4.3. Circular Dichroism Spectroscopy

CD spectra were recorded three times independently at 20 °C at a rate of 50 nm/min from 200–320 nm. The cell path length was 0.1 cm. The duplex nucleic-acid concentration used was 4 μM, and the samples were prepared in the same method described in the UV melting experiments.

### 4.4. Nuclease Stability Experiments

Degradation of oligonucleotides (0.0175 nM) by snake venom phosphodiesterase (SVPDE; 0.02 μg/mL) was performed in a buffer (pH 8.0) containing 10 mM MgCl_2_, 50 mM Tris-HCl at 37 °C. Aliquots were taken at several time points and quenched by methanol to deactivate the nuclease. The timed reaction mixtures were analyzed by HPLC to evaluate the amount of intact oligonucleotides remaining. The percentage of intact oligonucleotide in each sample was calculated and plotted against the time of exposure to get the oligonucleotide degradation profile with time.

## Figures and Tables

**Table 1:** Melting temperature (*T*_m_) values (°C) of the duplexes formed by 2′-F,4′-*C*-OMe–arabinouridine (araU) (**X**) and 2′-F–araU (**Y**) modified DNA with their complementary single-stranded DNA and RNA (ssDNA and ssRNA) *^a^*. ON—oligonucleotide.

#	Sequence (5′–3′)	ssRNA		ssDNA		RNA Selectivity
		*T*_m_	Δ*T*_m_	*T*_m_	Δ*T*_m_ *^b^*	Δ*T*_m_ (RNA) − Δ*T*_m_ (DNA)
**ON1**	GCGTTTTTTGCT	47.5		51.7		
**ON2**	GCGTT**X**TTTGCT	47.0	−0.5	48.4	−3.3	2.8
**ON3**	GCGTT**Y**TTTGCT	46.3	−1.2	51.2	−0.5	−0.7
**ON4**	GCGTT**X**T**X**TGCT	46.3	−1.2	46.1	−5.6	4.4
**ON5**	GCG**X**T**X**T**X**TGCT	42.9	−4.6	ND *^c^*		
**ON6**	GCGTTGTTTGCT	50.4		56.6		
**ON7**	GCGT**X**GTTTGCT	52.0	1.6	52.5	−4.1	5.7
**ON8**	GCGTTG**X**TTGCT	49.5	−0.9	51.2	−5.4	4.5
**ON9**	GCGTTATTTGCT	45.6		51.3		
**ON10**	GCGT**X**ATTTGCT	48.2	2.6	47.9	−3.4	6.0
**ON11**	GCGTTA**X**TTGCT	44.6	−1.0	46.5	−4.8	3.7

*^a^* The ultraviolet (UV) melting experiments were carried out in 10 mM sodium phosphate buffer (pH 7.2) containing 100 mM NaCl at a rate of 1 °C/min at 260 nm. The concentration of the oligonucleotide was 4 μM for each strand. The sequences of target DNA and RNA complements were 5′–d(AGCAAAAAACGC)–3′ and 5′–r(AGCAAAAAACGC)–3′ for **ON1**–**ON5**, 5′–d(AGCAAACAACGC)–3′ and 5′–r(AGCAAACAACGC)–3′ for **ON6**–**ON8**, and 5′–d(AGCAAATAACGC)–3′ and 5′–r(AGCAAAUAACGC)–3′ for **ON9**–**ON11**. *^b^* Δ*T*_m_ = *T*_m(modified)_ − *T*_m(unmodified)_; *^c^* ND: not determined as typical “S”-shape melting curve was not formed.

## Supplementary Materials

The following are available online: Table S1: The mass spectra data and HPLC purity of modified oligonucleotides; Table S2: ^1^H–^1^H and ^1^H–^19^F coupling constant values for 2′-F,4′-*C*-OMe–araU and 2′-F–araU; NMR spectra of intermediates and phosphoramidite; HPLC charts and MS spectra of modified oligonucleotides.
