# Supplementary material for: Synthesis and Antisense Properties of 2′β-F-Arabinouridine Modified Oligonucleotides with 4′-C-OMe Substituent
Source: Molecules. 2018 Sep 17;23(9):2374. doi: 10.3390/molecules23092374 (PMC6225415; doi:10.3390/molecules23092374)

Supplementary Materials

Synthesis and antisense properties of 2'β-F-arabinouridine modified oligonucleotides with 4'-*C*-OMe substituent

Xiao-Yang He [1,*,†], Jing Wang [2,†], Dan-Dan Lu [1] and Sheng-Qi Wang [1,*]

[1] Beijing Institute of Radiation Medicine, Beijing 100850, China; lu_dandan@163.com
[2] School of Pharmaceutical Sciences, Zhengzhou University, Zhengzhou 450001, China; anna_wj@163.com
* Correspondence: hexiaoyang@aliyun.com (X.-Y.H.); sqwang@bmi.ac.cn (S.-Q.W.); Tel.: +86-10-6693-1423.
† These authors contributed equally to this work.

Content: **PP.**

Table S1 The mass spectra data and HPLC purity of modified oligonucleotides S2

Table S2 ^{1}H-^{1}H and ^{1}H-^{19}F coupling constant values for 2′-F,4′-*C*-OMe-araU and 2′-F-araU S2

NMR spectra of intermediates and phosphoramidite S3-S10

HPLC charts and MS spectra of modified oligonucleotides S11-S18

Table s1 The mass spectra data and HPLC purity of modified oligonucleotides, (**X** = 2'-F,4'-*C*-OMe-araU, **Y** = 2'-F-araU, **Ts** = 3'-phosphorothioate-T).

#	Sequence	UV purity/%	Mass Calcd. $[M-H]^-$	Mass Found $[M-H]^-$
**ON2**	5'-d(GCGTT**X**TTTGCT)-3'	95.49	3666.4	3666.4
**ON3**	5'-d(GCGTT**Y**TTTGCT)-3'	97.33	3636.3	3639.3
**ON4**	5'-d(GCGTT**X**T**X**TGCT)-3'	97.67	3700.3	3705.0
**ON5**	5'-d(GCG**X**T**X**T**X**TGCT)-3'	95.79	3734.3	3739.1
**ON7**	5'-d(GCGT**X**GTTTGCT)-3'	99.75	3692.4	3694.7
**ON8**	5'-d(GCGTTG**X**TTGCT)-3'	95.18	3692.4	3693.0
**ON10**	5'-d(GCGT**X**ATTTGCT)-3'	97.84	3676.4	3676.4
**ON11**	5'-d(GCGTTA**X**TTGCT)-3'	98.56	3676.4	3677.4
**ON12**	5'-d(TTTTTTTT**X**T)-3'	99.28	3013.0	3016.0
**ON13**	5'-d(TTTTTTTT**Y**T)-3'	99.22	3058.0	3055.5
**ON14**	5'-d(TTTTTTTT**Ts**T)-3'	99.70	2995.0	2998.7

Table s2 ^{1}H-^{1}H and ^{1}H-^{19}F coupling constant values for 2'-F,4'-*C*-OMe-araU and 2'-F-araU at 25 °C in D_2O (400 MHz).

J (Hz) (±0.2)	2'-F,4'-*C*-OMe-araU	2'-F-araU
H1'-H2''	5.8	4.0
H1'-F2''	9.0	17.5
H2''-H3'	5.6	3.0
H3'-F2'	24.0	21.5
H3'-X4'	-	5.0

NMR spectra of intermediates and phosphoramidite

Compound 2

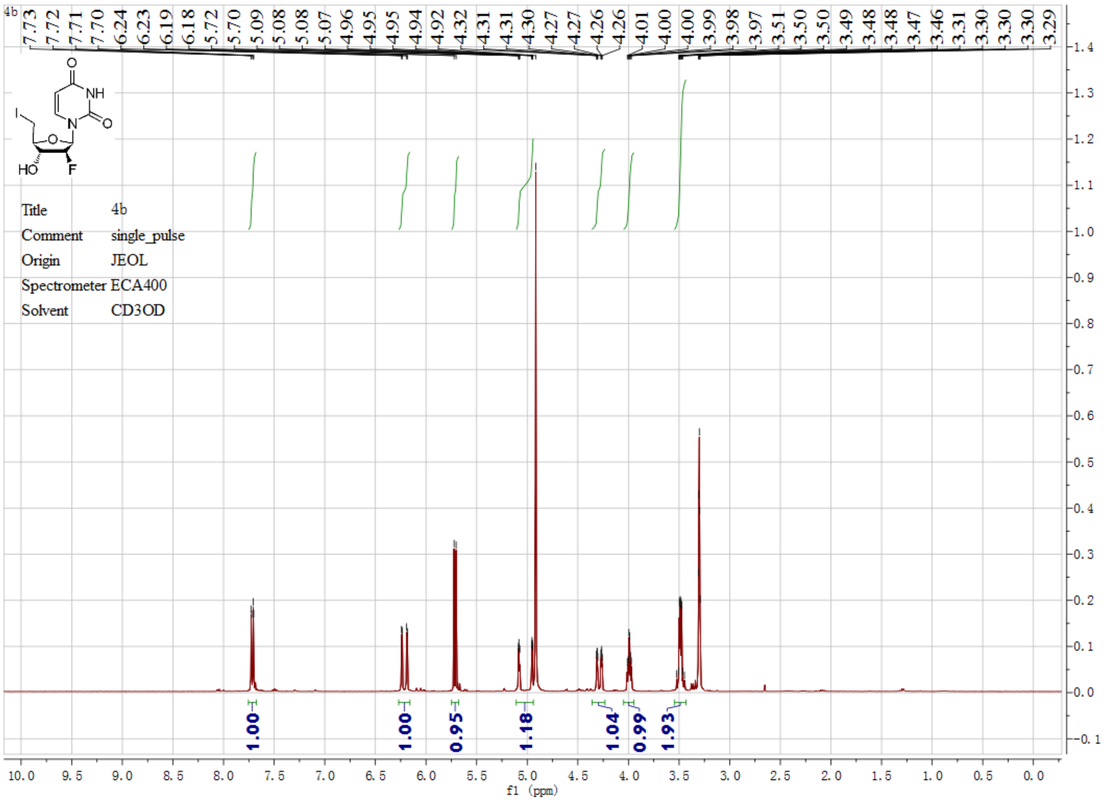

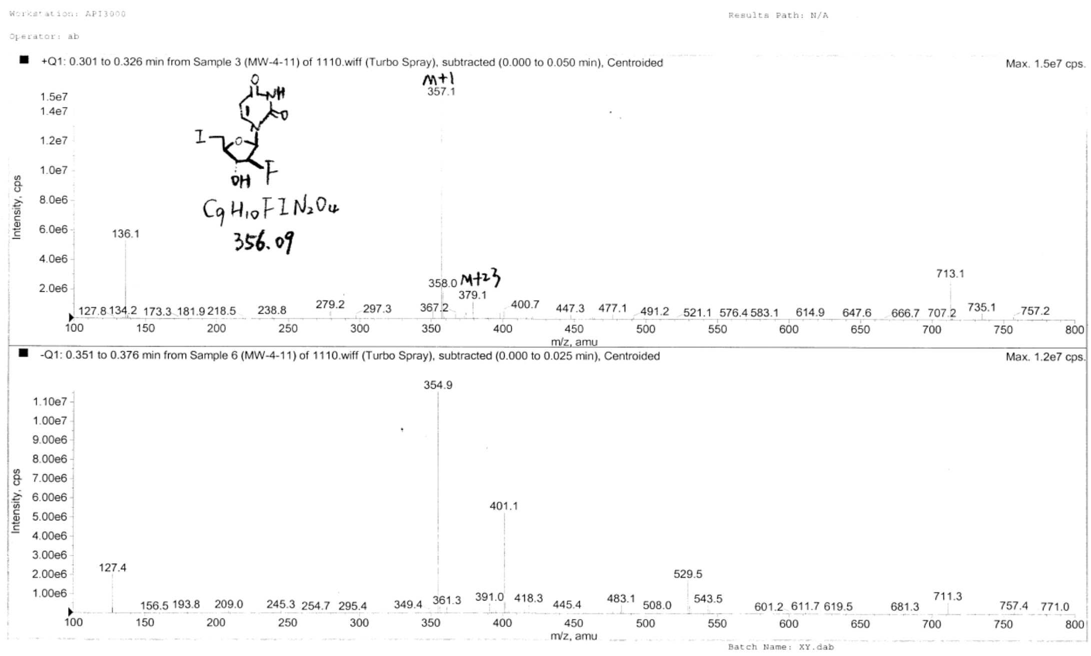

Compound 3

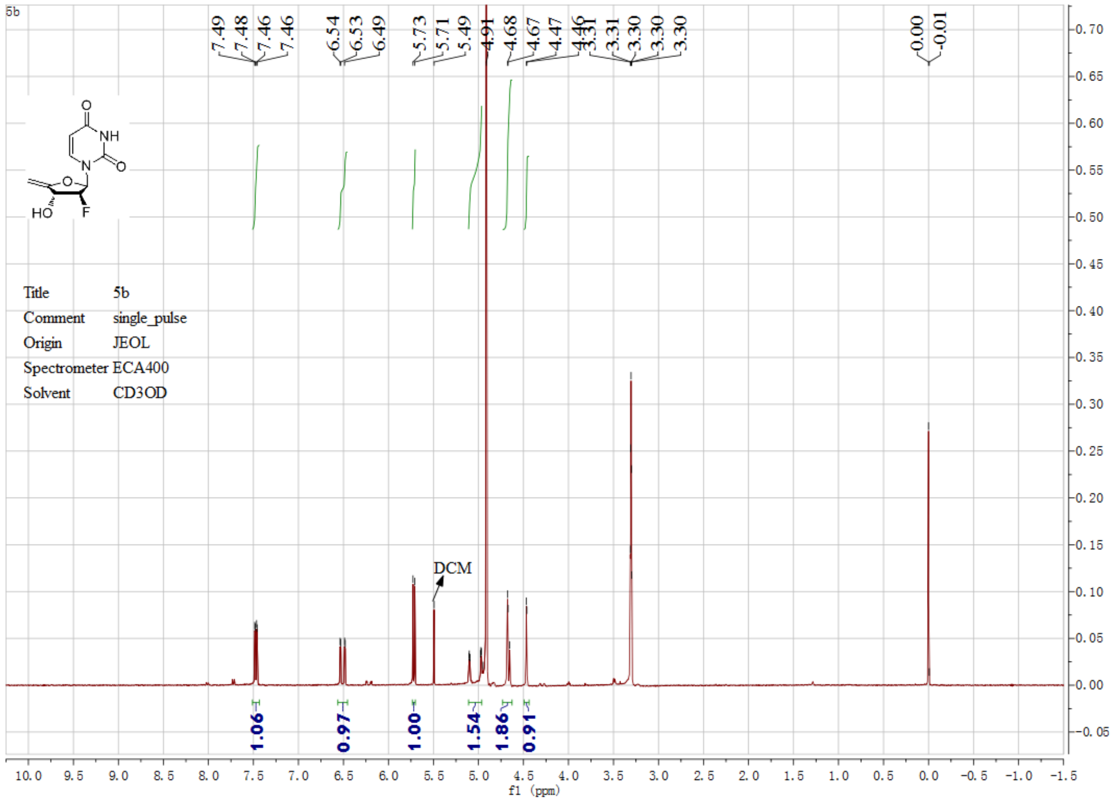

5b
7.49
7.48
7.46
7.46
6.54
6.53
6.49
5.73
5.71
5.49
4.91
4.68
4.67
4.47
4.46
3.31
3.31
3.30
3.30
3.30
0.00
-0.01
Title 5b
Comment single_pulse
Origin JEOL
Spectrometer ECA400
Solvent CD3OD
DCM
1.06
0.97
1.00
1.54
1.86
0.91
f1 (ppm)

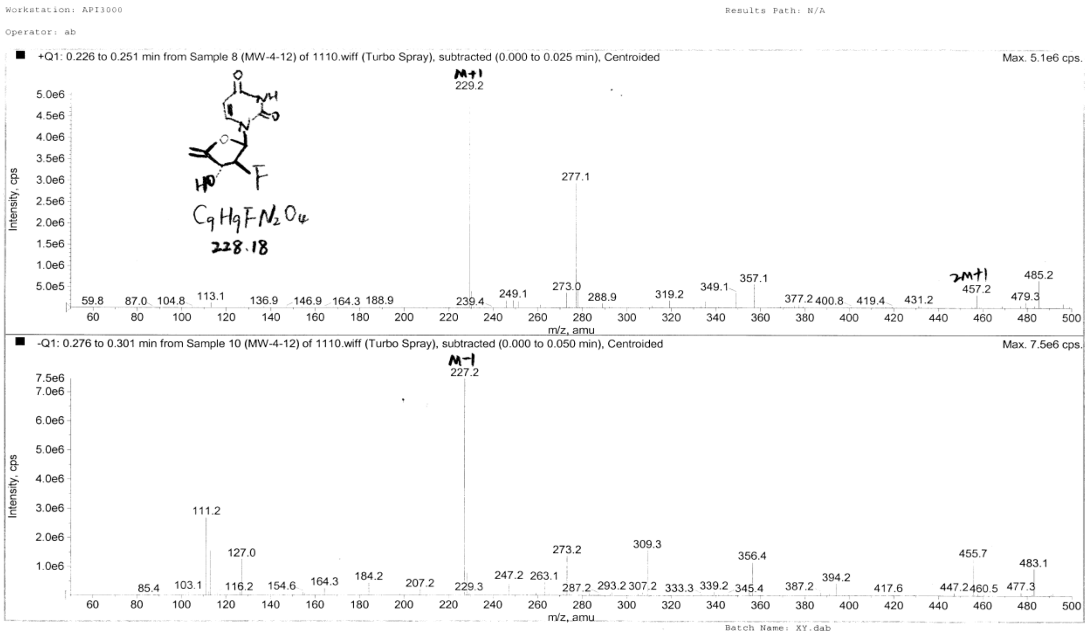

Workstation: API3000
Results Path: N/A
Operator: ab
+Q1: 0.226 to 0.251 min from Sample 8 (MW-4-12) of 1110.wiff (Turbo Spray), subtracted (0.000 to 0.025 min), Centroided
Max. 5.1e6 cps.
M+1
229.2
277.1
2M+1
457.2
$C_9H_9FN_2O_4$
228.18
Intensity, cps
m/z, amu
-Q1: 0.276 to 0.301 min from Sample 10 (MW-4-12) of 1110.wiff (Turbo Spray), subtracted (0.000 to 0.050 min), Centroided
Max. 7.5e6 cps.
M-1
227.2
111.2
Batch Name: XY.dab

Compound 4

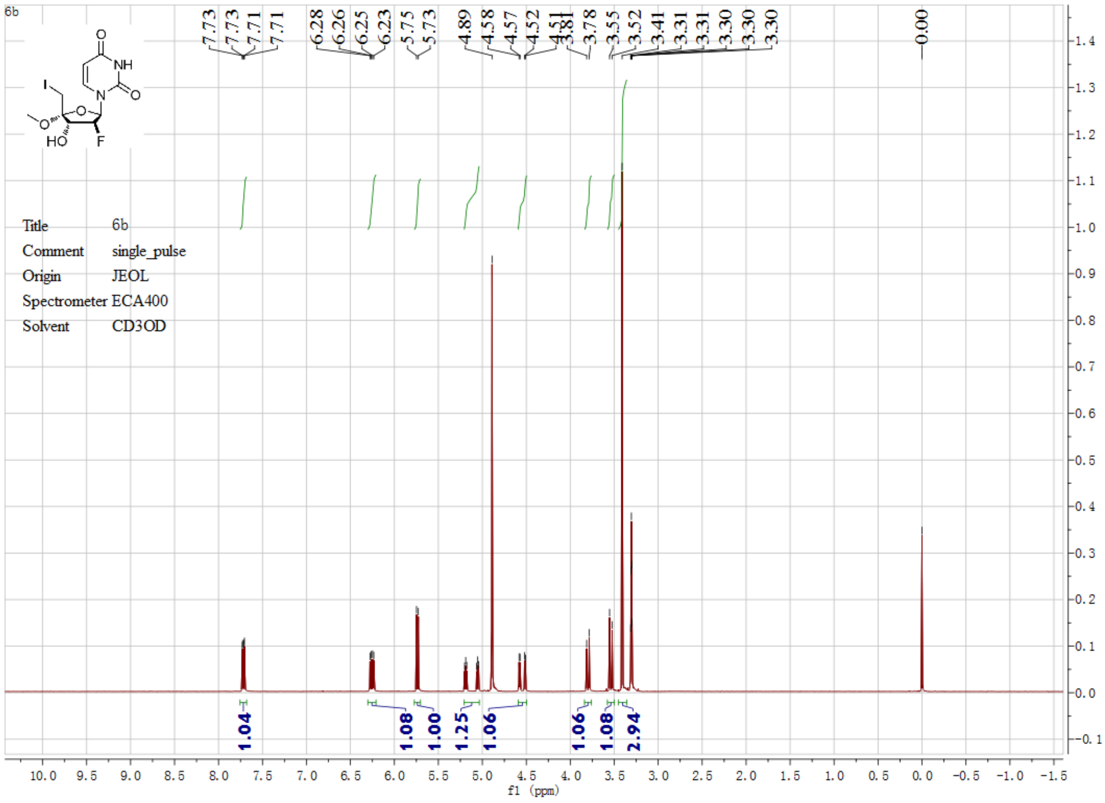

6b
Title 6b
Comment single_pulse
Origin JEOL
Spectrometer ECA400
Solvent CD3OD
f1 (ppm)

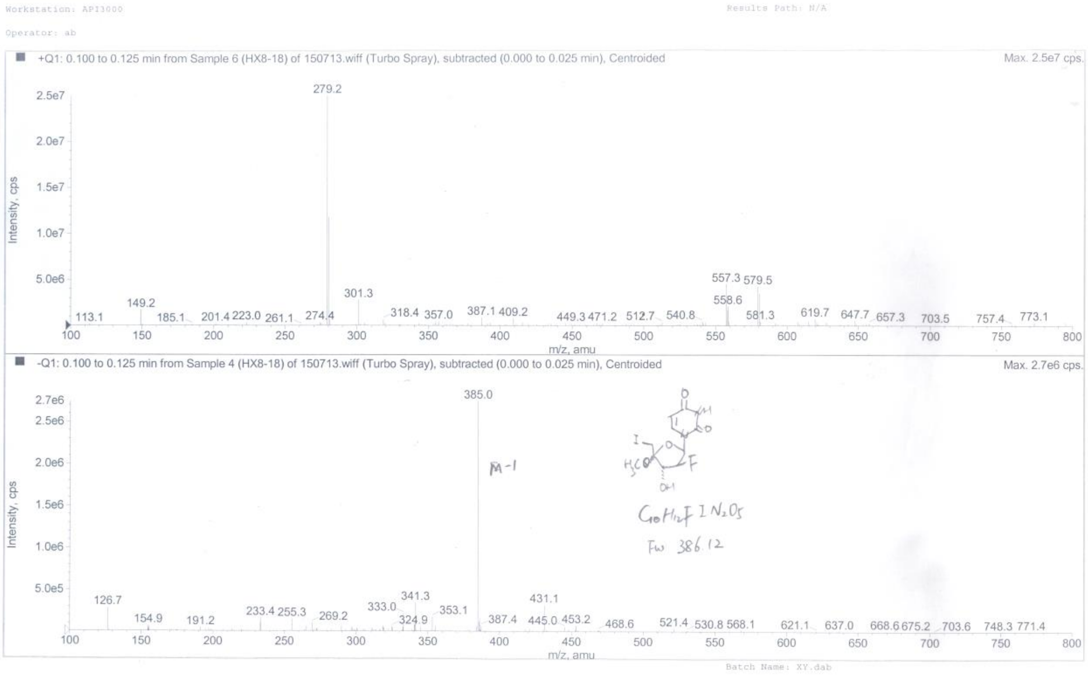

Workstation: API3000
Results Path: N/A
Operator: ab
+Q1: 0.100 to 0.125 min from Sample 6 (HX8-18) of 150713.wiff (Turbo Spray), subtracted (0.000 to 0.025 min), Centroided
Max. 2.5e7 cps.
-Q1: 0.100 to 0.125 min from Sample 4 (HX8-18) of 150713.wiff (Turbo Spray), subtracted (0.000 to 0.025 min), Centroided
Max. 2.7e6 cps.
Intensity, cps
m/z, amu
M-1
Batch Name: XY.dab

Compound 5

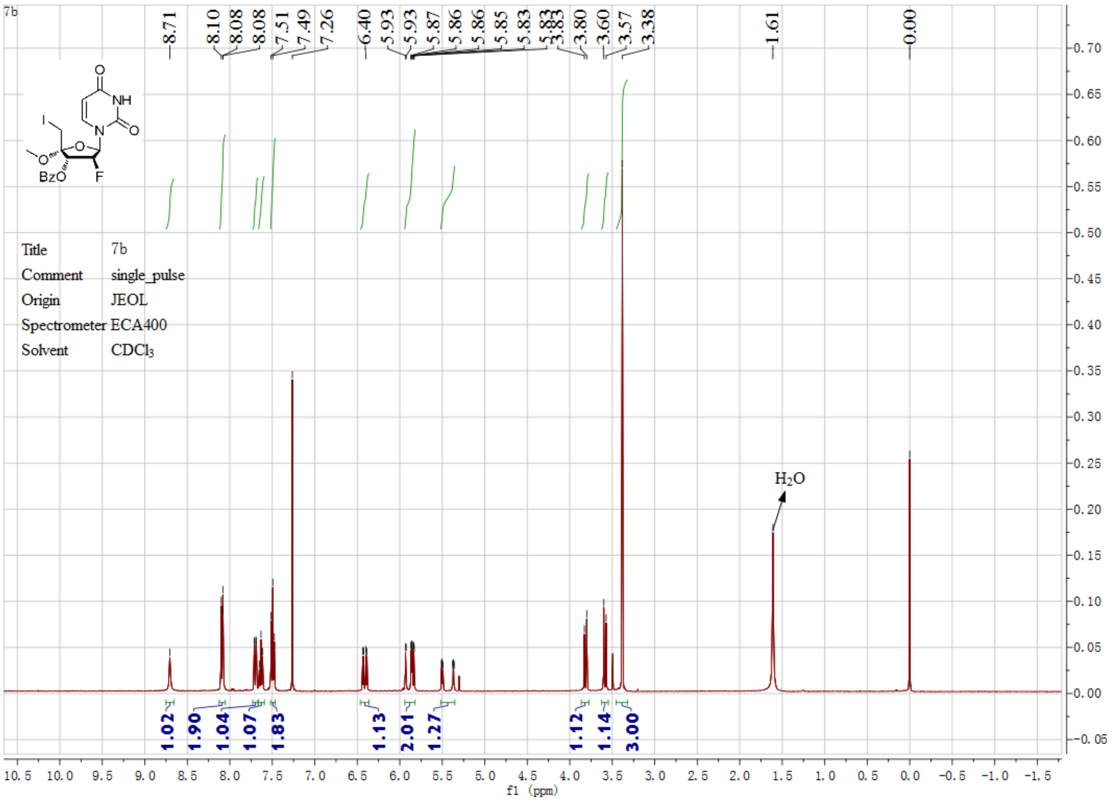

Qualitative Analysis Report

**Data Filename**	4277.d	**Sample Name**	HX-8-19A
**Instrument Name**	TOF G6230A	**Acquired Time**	2018-08-15
**Acq Method**	YCL.M	**Acquired SW**	6200 series TOF/6500 series
**IRM Calibration Status**	**Success**		
**User Chormatograms**			

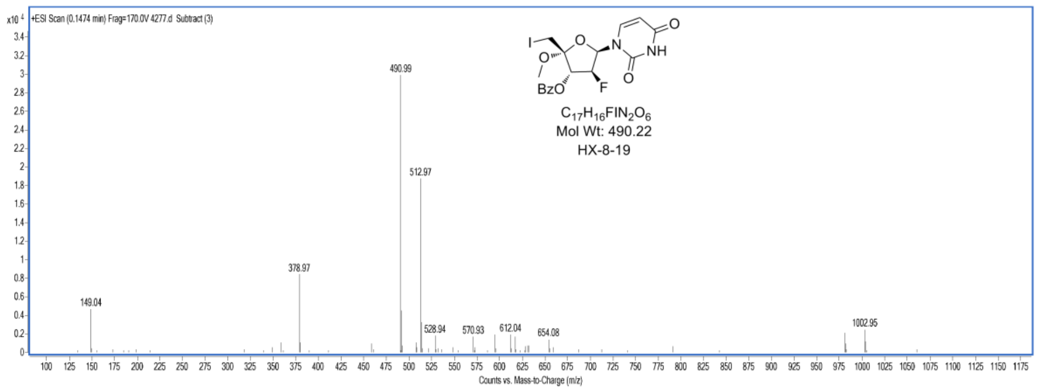

Compound 6

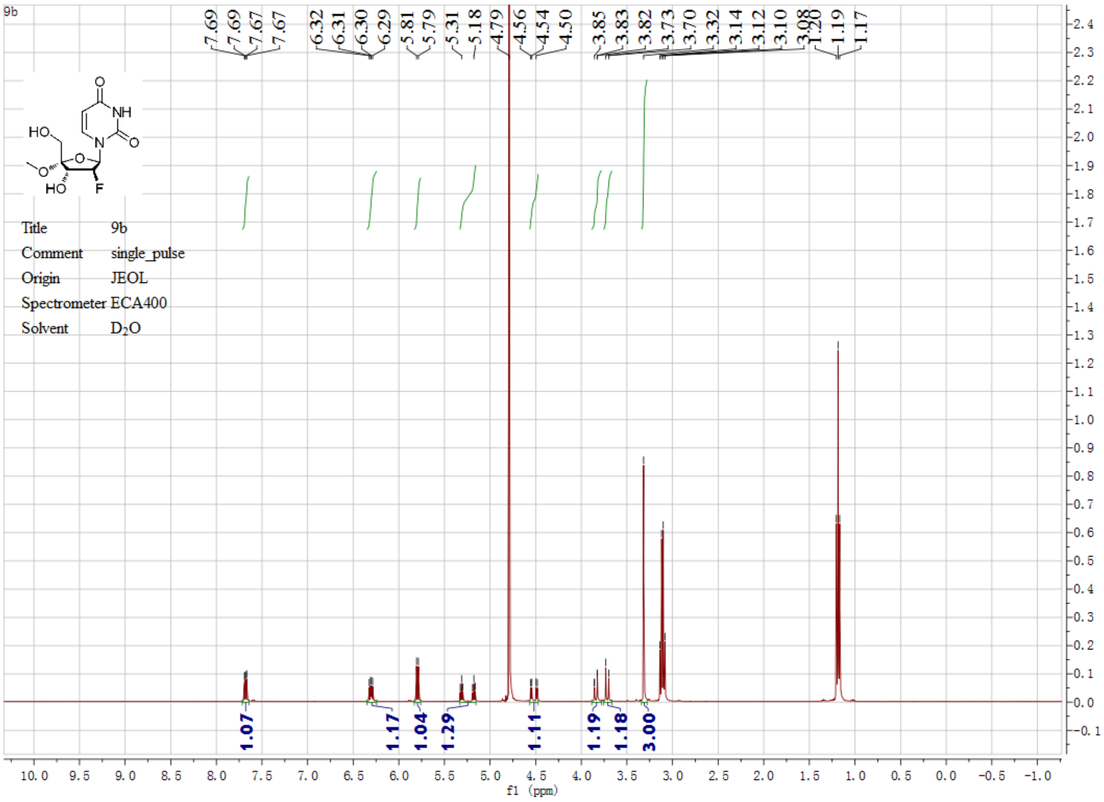

Qualitative Analysis Report

Data Filename	2720.d	Sample Name	HX-8-20
Instrument Name	TOF G6230A	Acquired Time	2015-09-29
Acq Method	YCL.M	Acquired SW	6200 series TOF/6500 series
IRM Calibration Status	Success		
User Chormatograms			

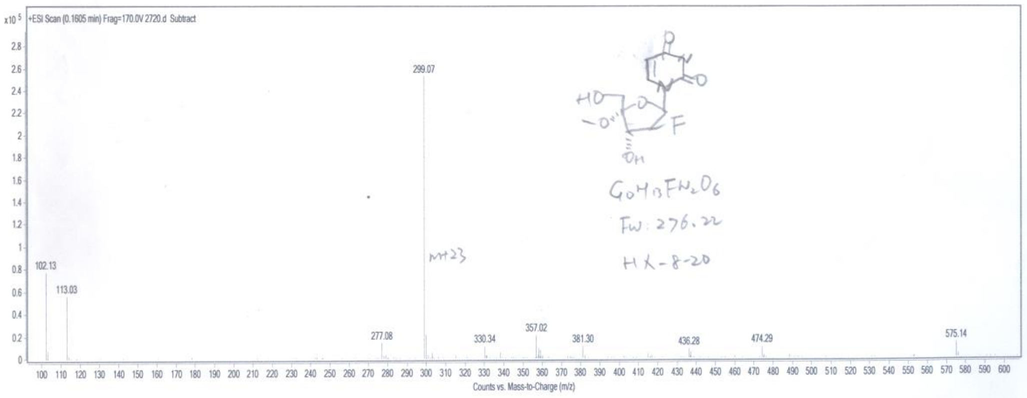

Compound 7

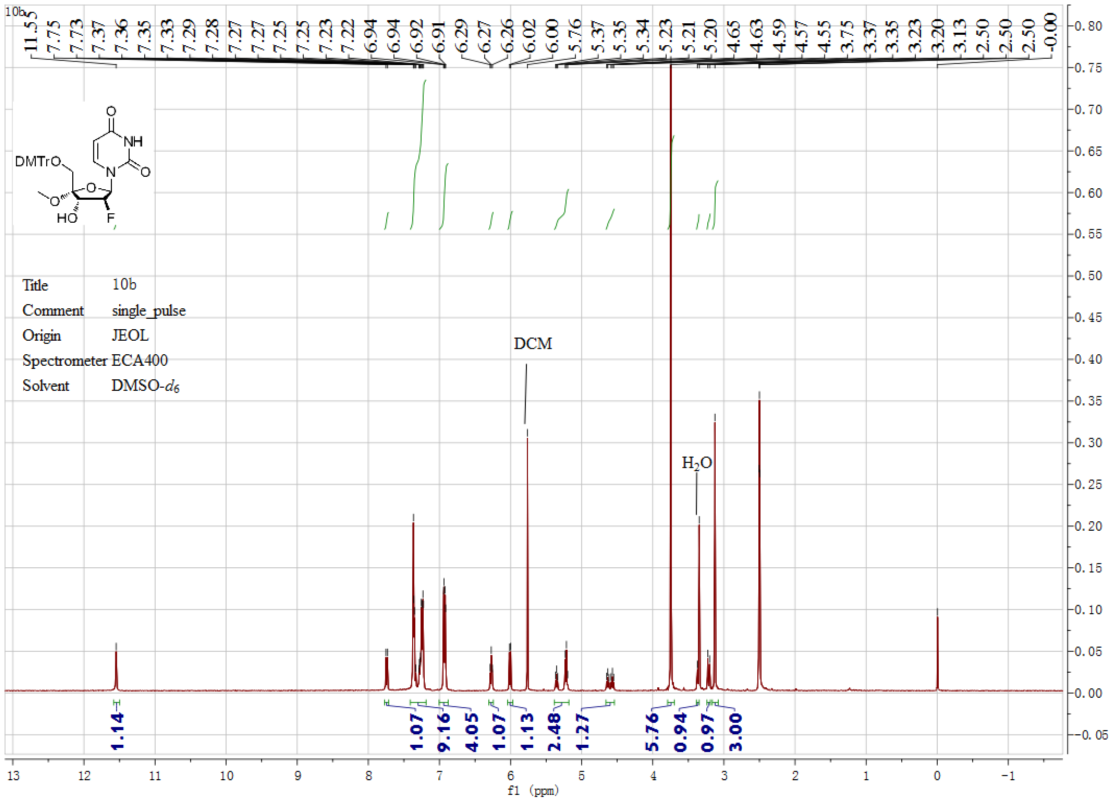

10b
DMTrO
NH
O
N
HO
F
Title 10b
Comment single_pulse
Origin JEOL
Spectrometer ECA400
Solvent DMSO-d_6
DCM
H_2O
1.14
1.07
9.16
4.05
1.07
1.13
2.48
1.27
5.76
0.94
0.97
3.00
f1 (ppm)

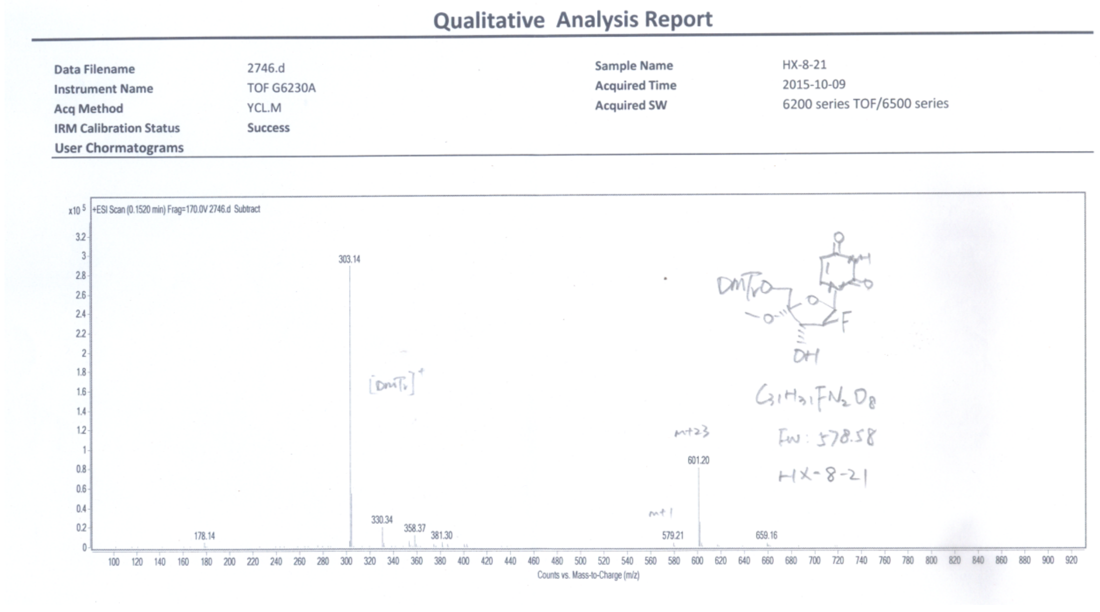

Qualitative Analysis Report
Data Filename 2746.d
Instrument Name TOF G6230A
Acq Method YCL.M
IRM Calibration Status Success
User Chormatograms
Sample Name HX-8-21
Acquired Time 2015-10-09
Acquired SW 6200 series TOF/6500 series
+ESI Scan (0.1520 min) Frag=170.0V 2746.d Subtract
303.14
[DMTr]+
330.34
358.37
381.30
178.14
579.21
601.20
659.16
Counts vs. Mass-to-Charge (m/z)
HX-8-21

Compound 8

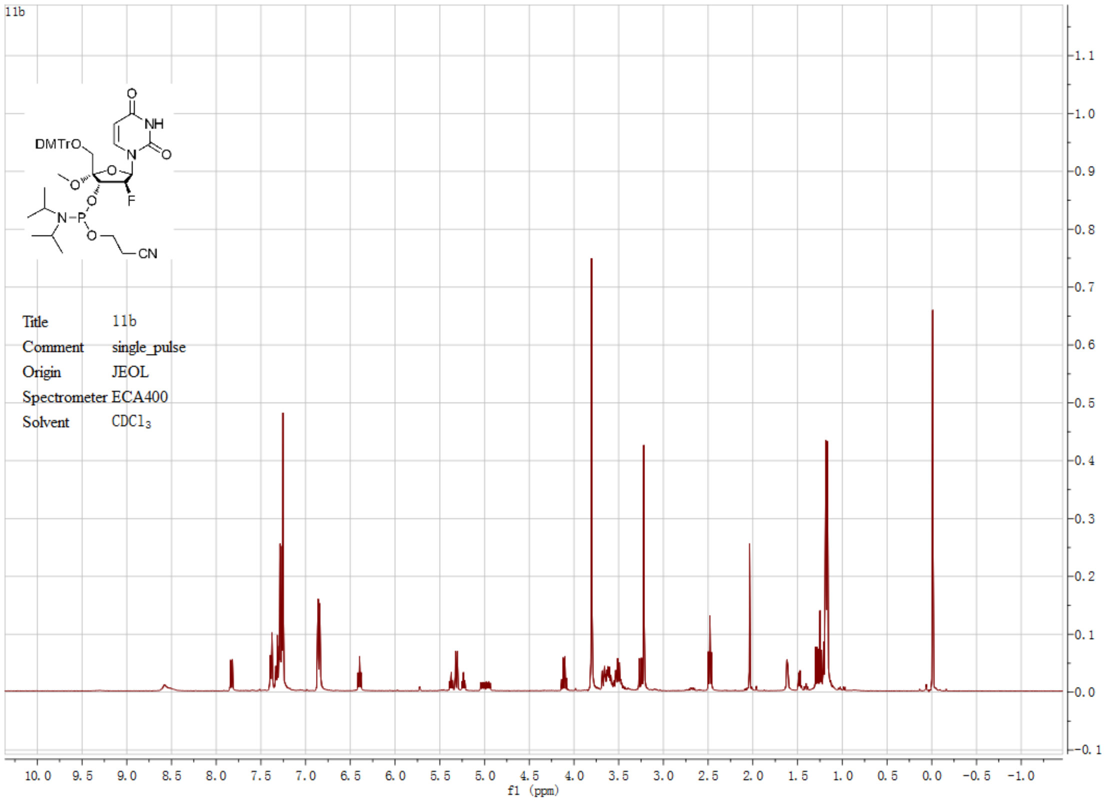
11b
DMTrO
NH
O
N
CN
F
Title 11b
Comment single_pulse
Origin JEOL
Spectrometer ECA400
Solvent CDCl3
f1 (ppm)

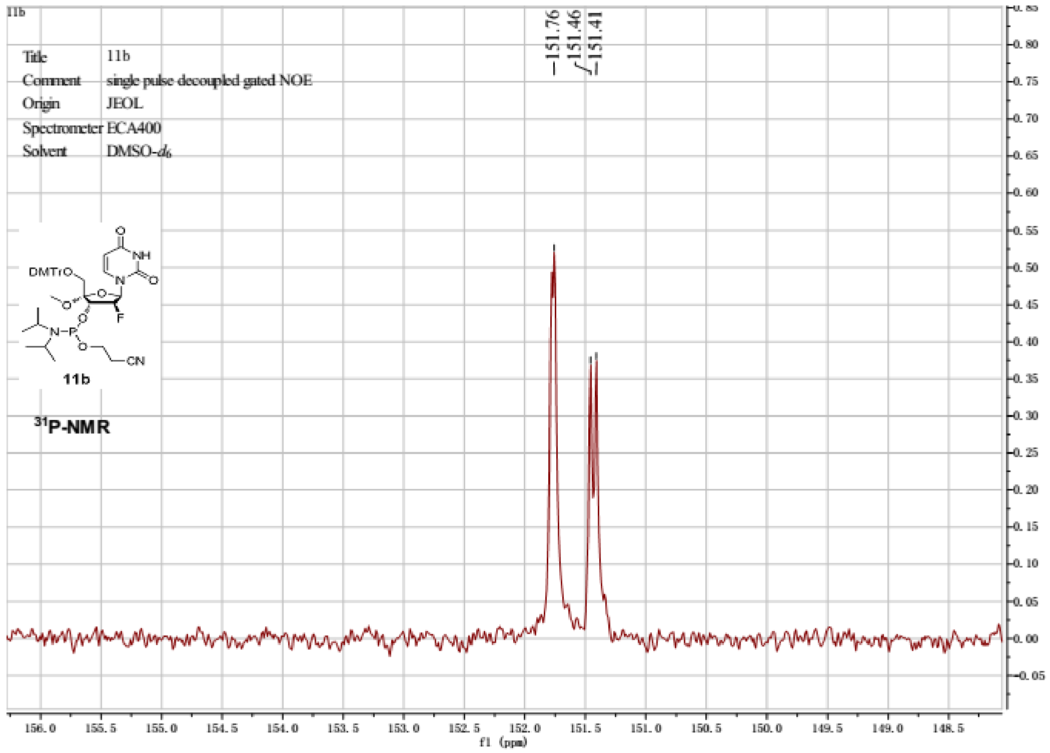
11b
Title 11b
Comment single pulse decoupled gated NOE
Origin JEOL
Spectrometer ECA400
Solvent DMSO-d6
−151.76
151.46
−151.41
DMTrO
NH
CN
F
11b
31P-NMR
f1 (ppm)

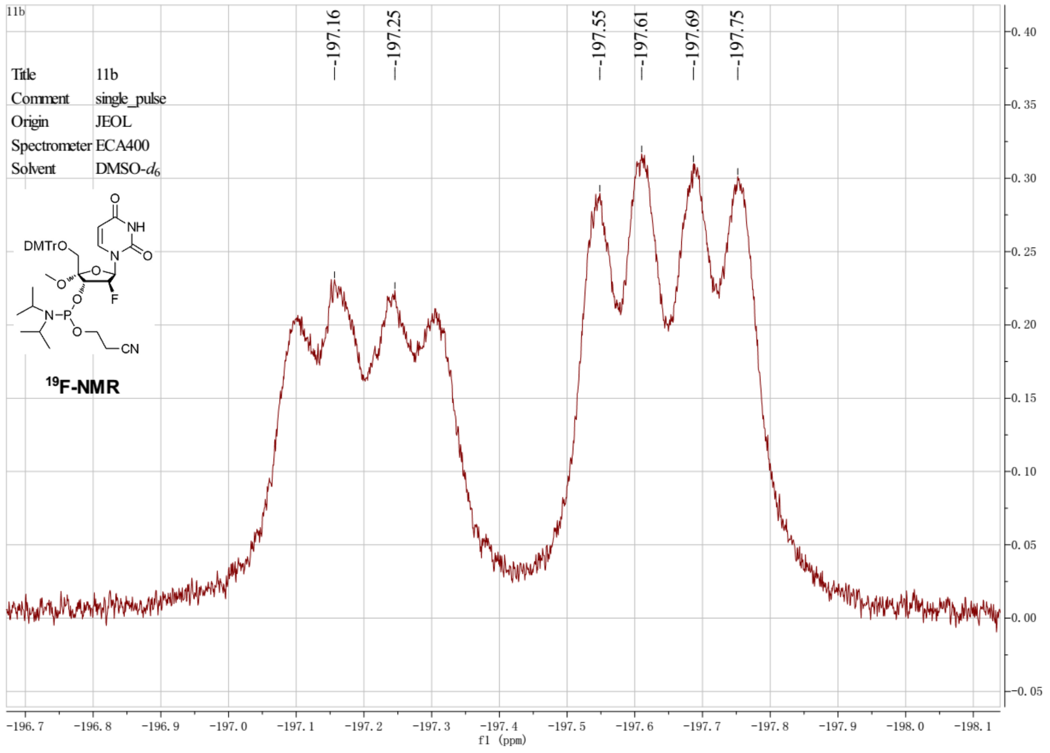

Qualitative Analysis Report

Data Filename	2357.d	Sample Name	WJ-26
Instrument Name	TOF G6230A	Acquired Time	2015-08-04
Acq Method	YCL.M	Acquired SW	6200 series TOF/6500 series
IRM Calibration Status	Success		
User Chormatograms			

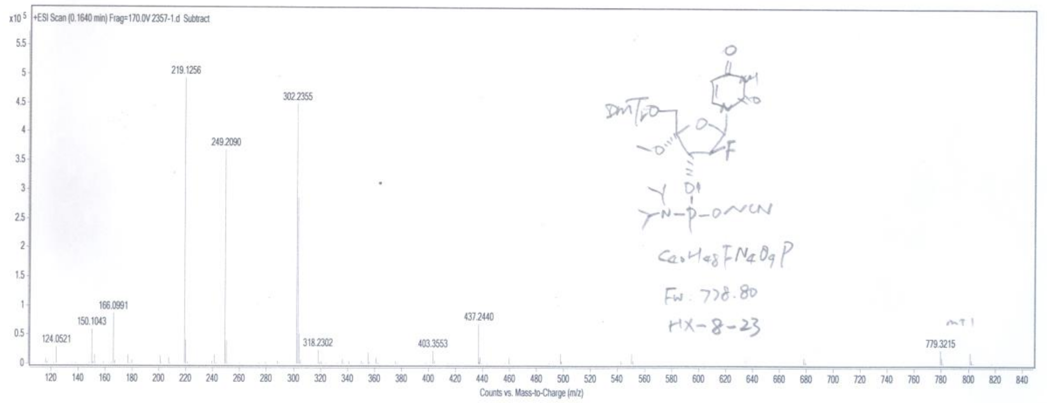

HPLC charts and MS spectra of modified oligonucleotides

ON2 5'-d(GCGTT**X**TTTGCT)-3' (**X**=2'-F-4'-OMe-araU)   Purity 95.49%

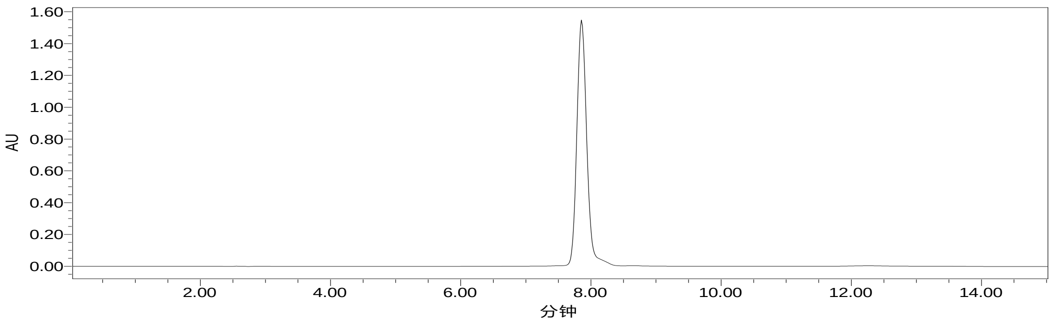

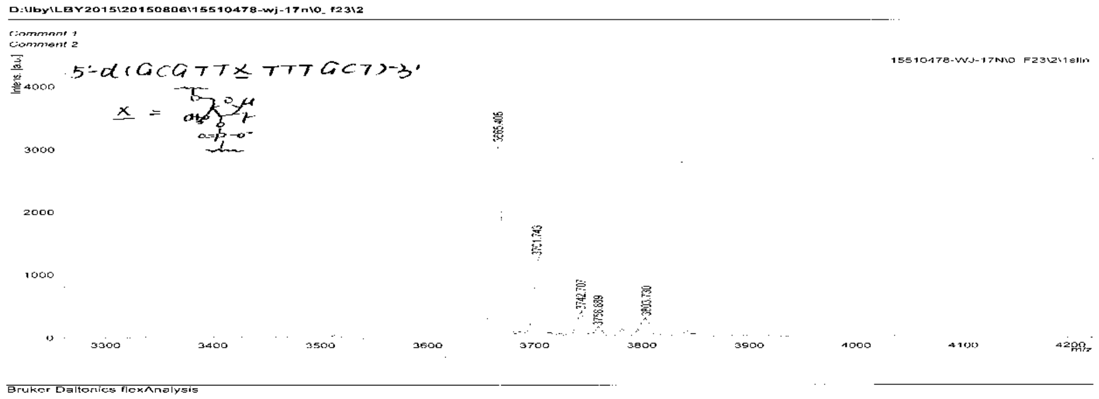

ON3 5'-d(GCGTT**Y**TTTGCT)-3' (**Y** = 2'-F-araU)   Purity 97.33%

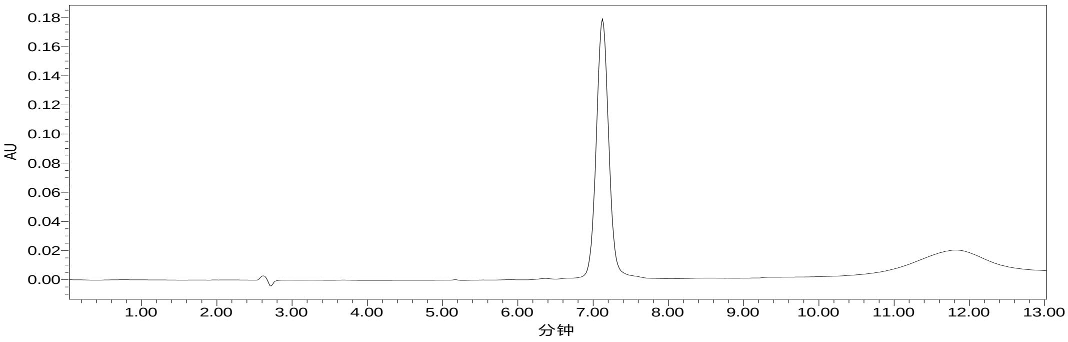

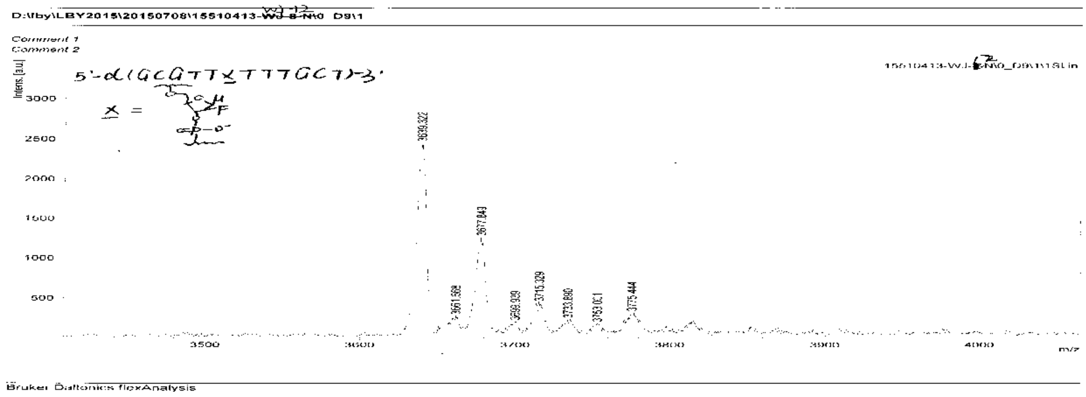

ON4 5'-d(GCGTT**X**T**X**TGCT)-3' (**X**=2'-F-4'-OMe-araU)   Purity 97.67%

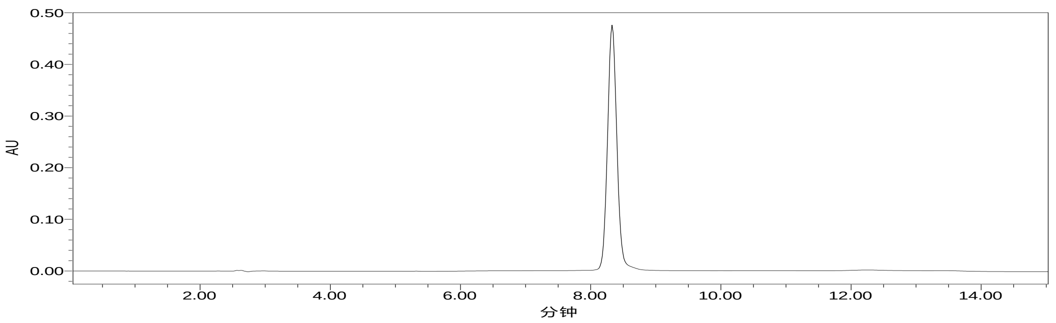

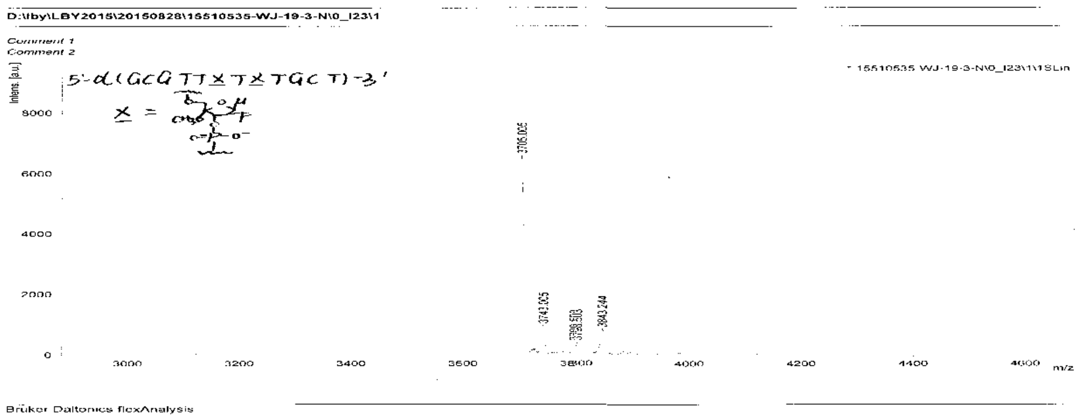

ON5 5'-d(GCG**X**T**X**T**X**TGCT)-3'(**X**=2'-F-4'-OMe-araU)   Purity 95.79%

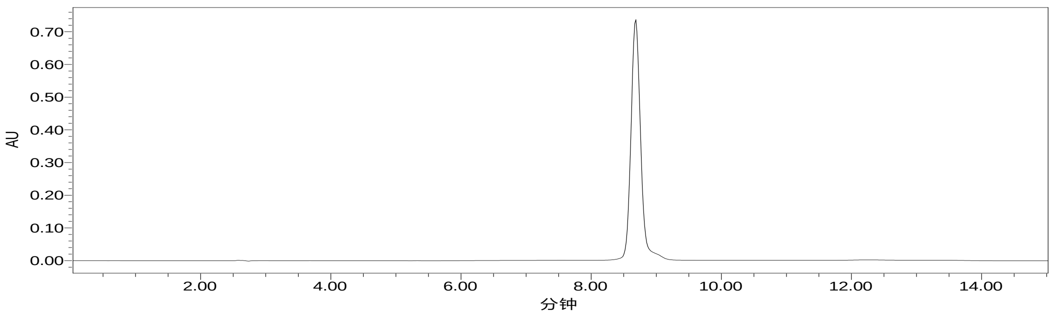

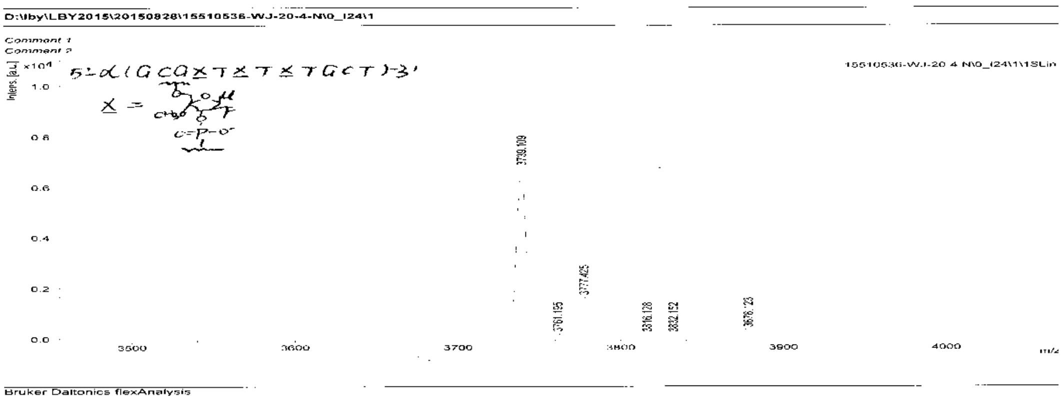

ON7 5'-d(GCGT**X**GTTTGCT)-3' (**X**=2'-F-4'-OMe-araU) purity: 99.75%

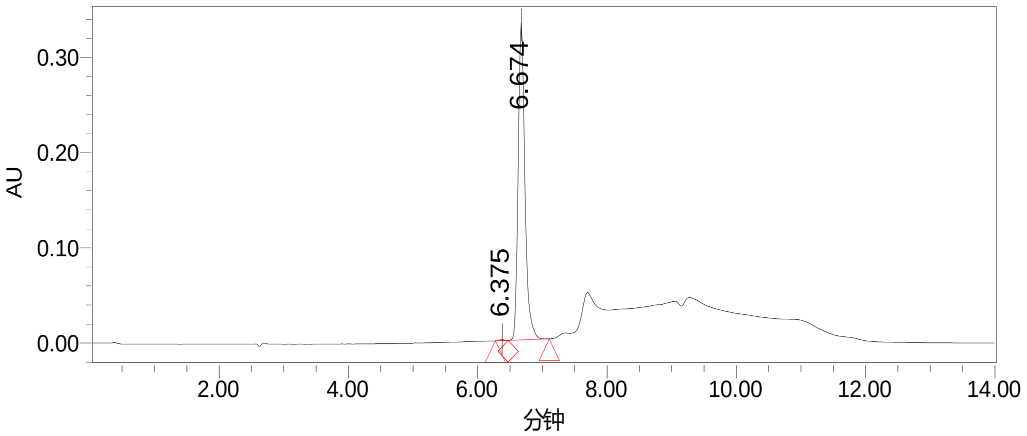

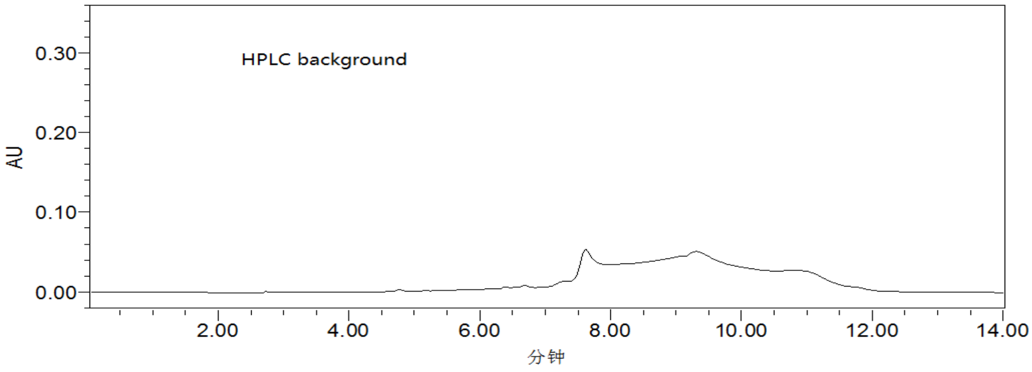
HPLC background
AU
0.30
0.20
0.10
0.00
2.00
4.00
6.00
8.00
10.00
12.00
14.00
分钟

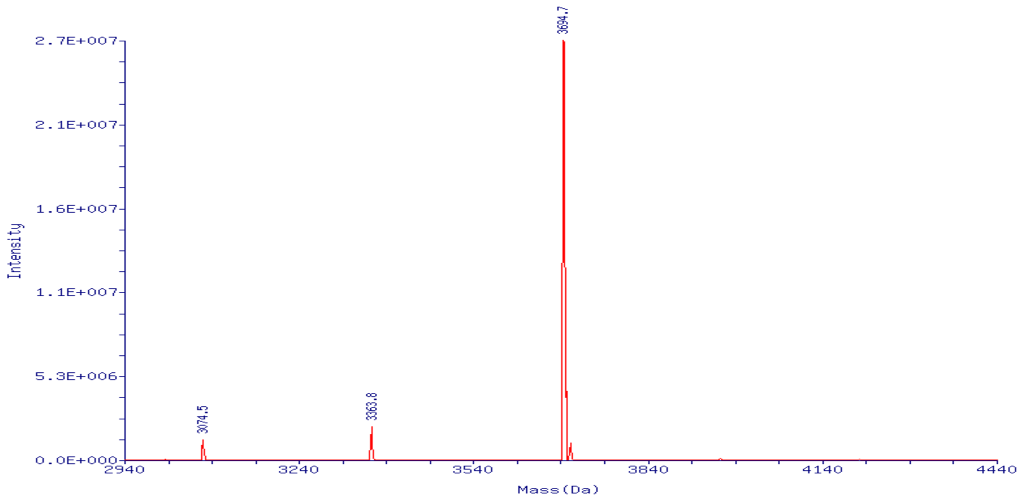
3694.7
3074.5
3363.8
2.7E+007
2.1E+007
1.6E+007
1.1E+007
5.3E+006
0.0E+000
Intensity
2940
3240
3540
3840
4140
4440
Mass(Da)

ON8 5'-d(GCGTTG**X**TTGCT)-3' (**X**=2'-F-4'-OMe-araU) purity: 95.18%

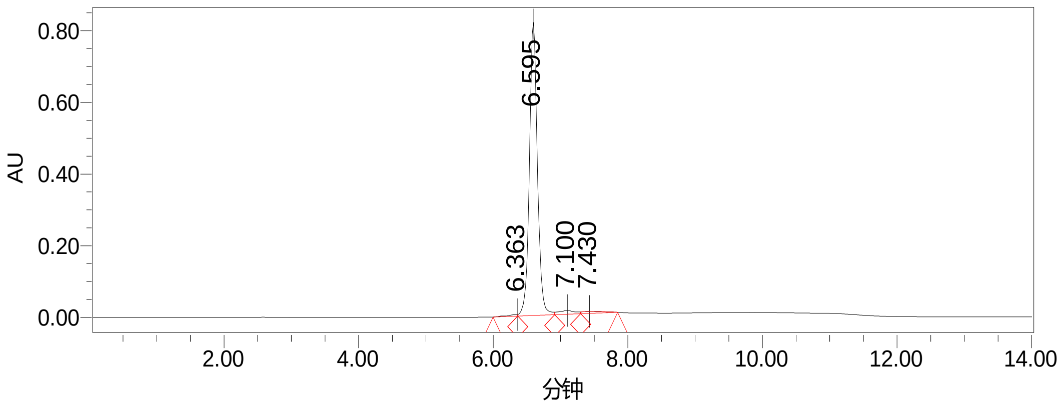

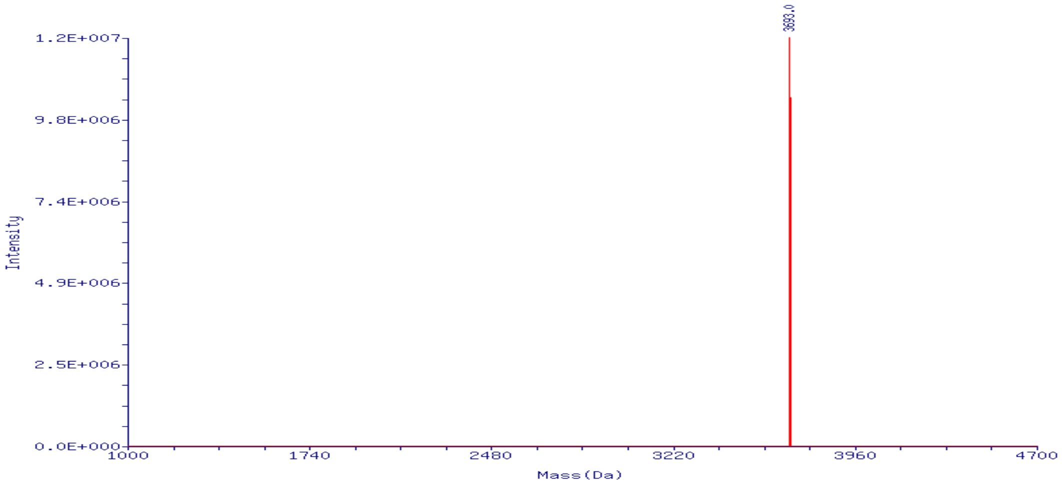

ON10 5'-d(GCGT**X**ATTTGCT)-3' (**X**=2'-F-4'-OMe-araU) purity: 97.84%

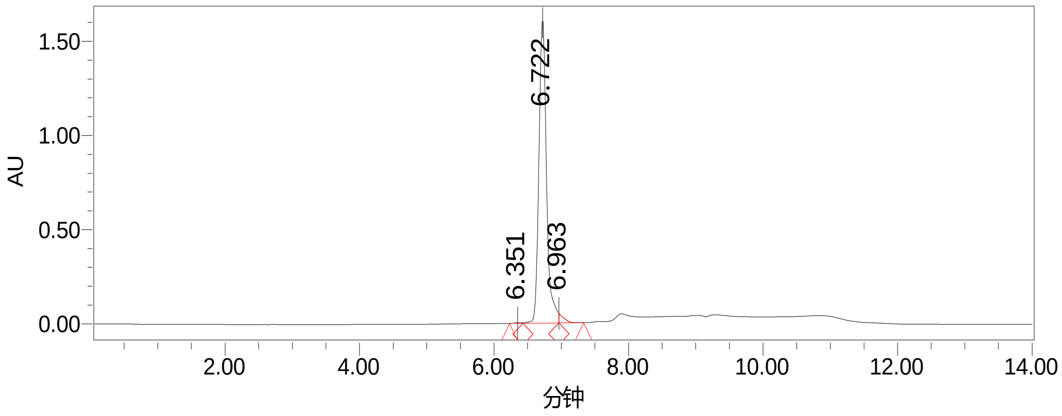

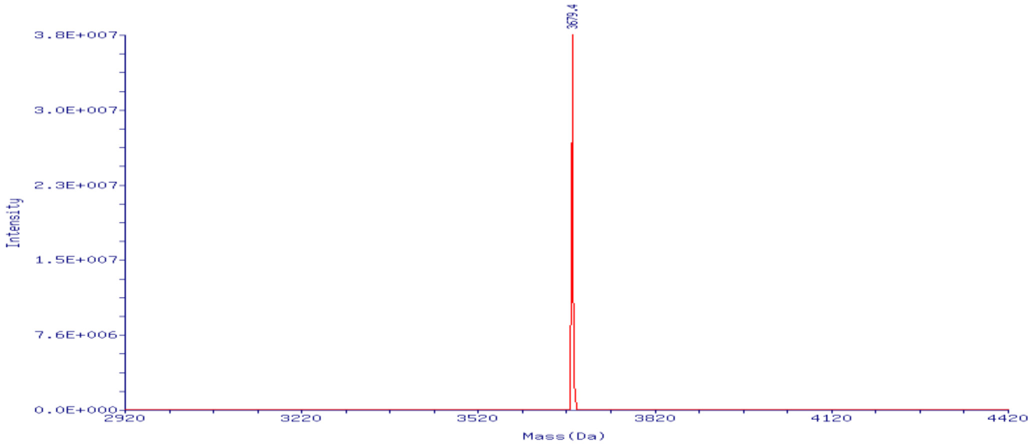

ON11 5'-d(GCGTTA**X**TTGCT)-3' (**X**=2'-F-4'-OMe-araU) purity: 98.56%

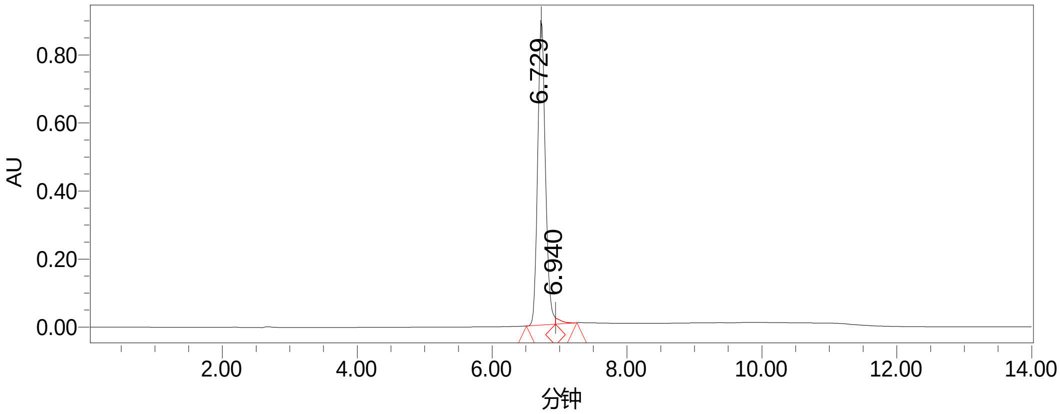

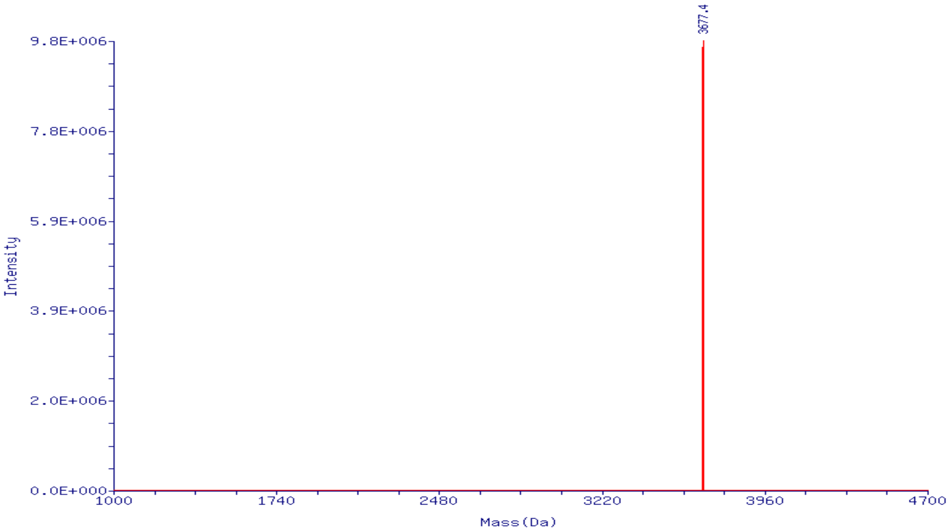

ON12 5'-d(TTTTTTTT**X**T)-3' (**X**=2'-F-4'-OMe-araU)   Purity 99.28%

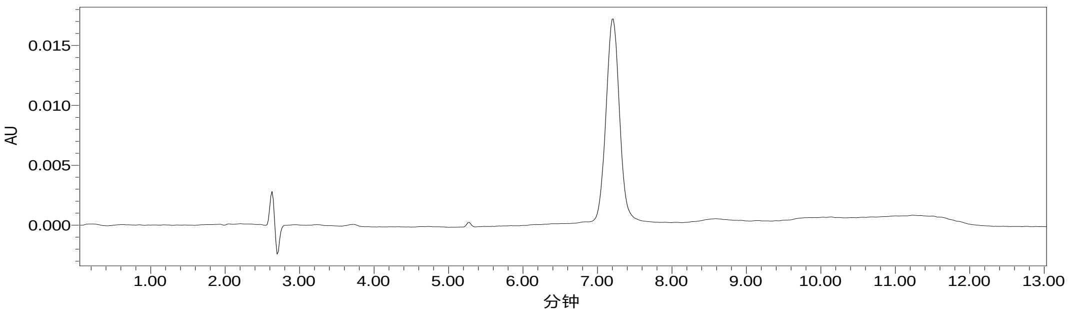

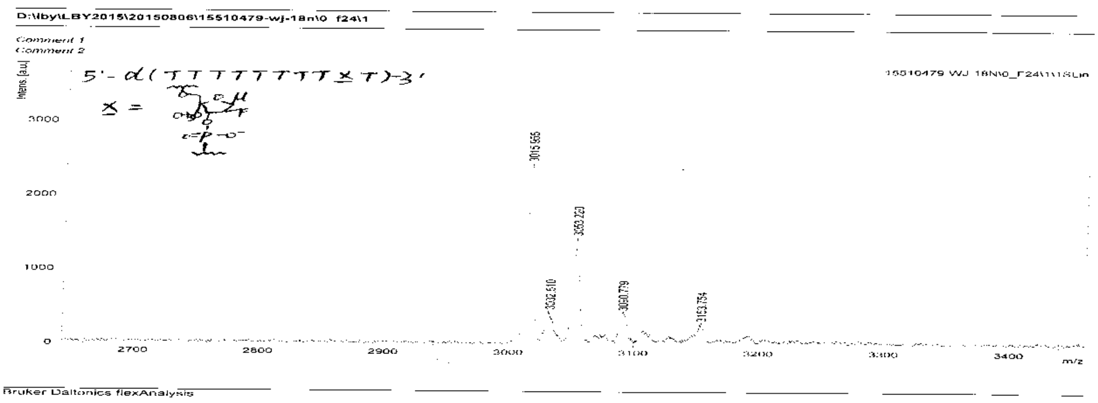

ON13 5'-d(TTTTTTTT**Y**T)-3' (**Y** = 2'-F-araU) Purity 99.22%

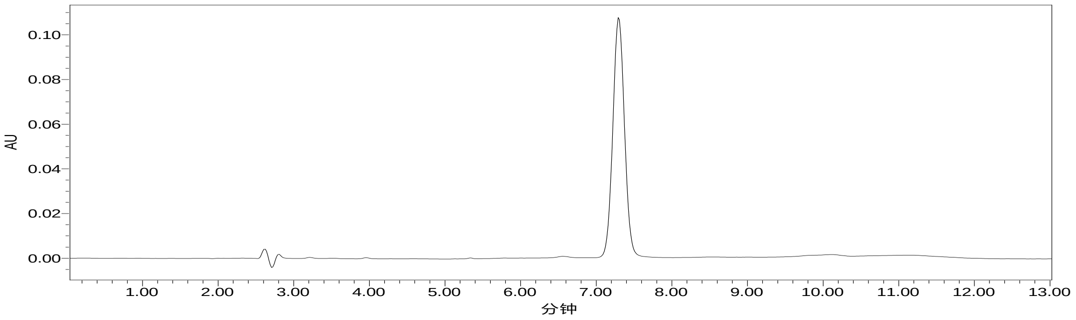

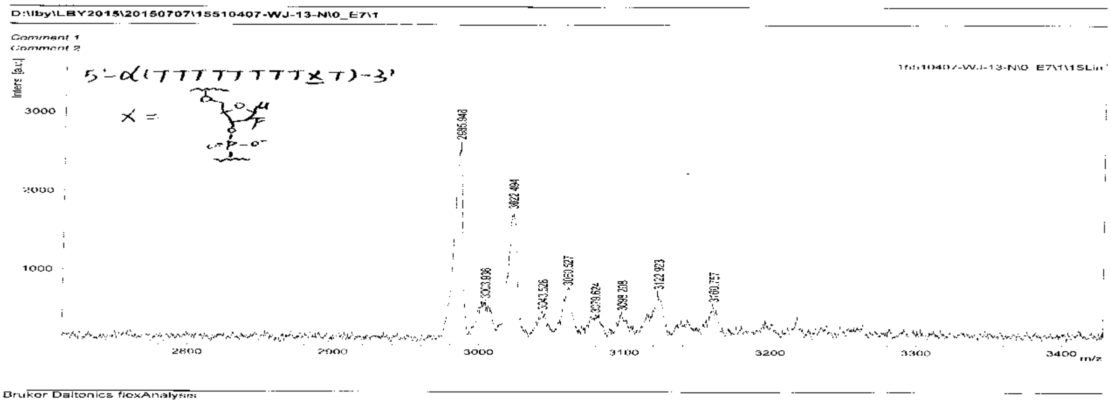

ON14 5'-d(TTTTTTTT**T**$_s$T)-3' (**Ts =** 3'-phosphorothioate-T) Purity 99.70%

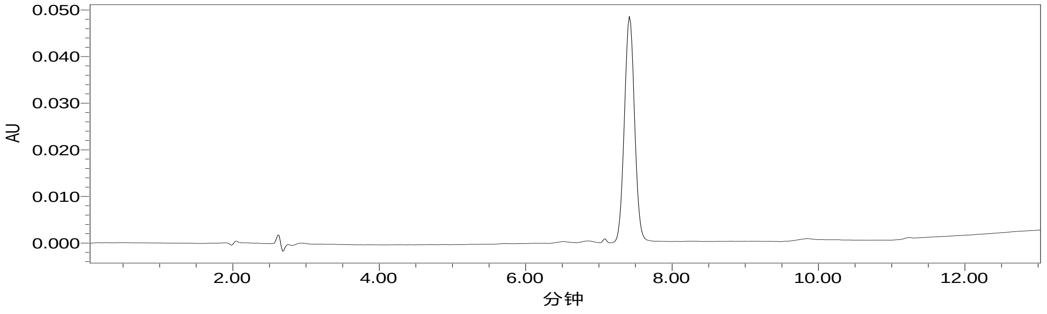

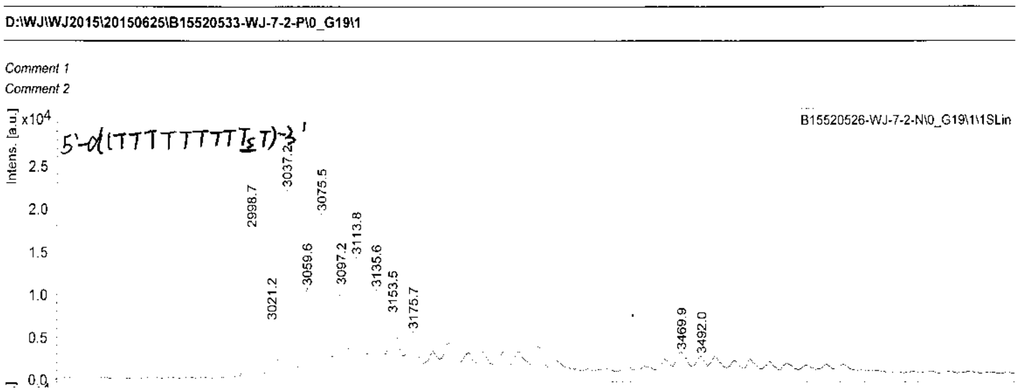

Supplement: Supplementary file 1 [file molecules-23-02374-s001.pdf]
